# Altered theta rhythm and hippocampal-cortical interactions underlie working memory deficits in a hyperglycemia risk factor model of Alzheimer’s disease

**DOI:** 10.1038/s42003-021-02558-4

**Published:** 2021-09-03

**Authors:** Ryan. A. Wirt, Lauren. A. Crew, Andrew. A. Ortiz, Adam. M. McNeela, Emmanuel Flores, Jefferson. W. Kinney, James M. Hyman

**Affiliations:** 1 Interdisciplinary Program in Neuroscience, University of Nevada Las Vegas, Las Vegas, NV USA; 2 Department of Brain Health, School of Integrated Health Sciences, University of Nevada, Las Vegas, NV USA; 3 Department of Psychology, University of Nevada Las Vegas, Las Vegas, NV USA

**Keywords:** Working memory, Neural ageing, Alzheimer's disease, Risk factors

## Abstract

*Diabetes mellitus* is a metabolic disease associated with dysregulated glucose and insulin levels and an increased risk of developing Alzheimer’s disease (AD) later in life. It is thought that chronic hyperglycemia leads to neuroinflammation and tau hyperphosphorylation in the hippocampus leading to cognitive decline, but effects on hippocampal network activity are unknown. A sustained hyperglycemic state was induced in otherwise healthy animals and subjects were then tested on a spatial delayed alternation task while recording from the hippocampus and anterior cingulate cortex (ACC). Hyperglycemic animals performed worse on long delay trials and had multiple electrophysiological differences throughout the task. We found increased delta power and decreased theta power in the hippocampus, which led to altered theta/delta ratios at the end of the delay period. Cross frequency coupling was significantly higher in multiple bands and delay period hippocampus-ACC theta coherence was elevated, revealing hypersynchrony. The highest coherence values appeared long delays on error trials for STZ animals, the opposite of what was observed in controls, where lower delay period coherence was associated with errors. Consistent with previous investigations, we found increases in phosphorylated tau in STZ animals’ hippocampus and cortex, which might account for the observed oscillatory and cognitive changes.

## Abstract (web summary)

To investigate the effects of chronic hyperglycemia on hippocampal network activity Wirt et al induced sustained hyperglycemia in rats and tested them in a spatial delayed alternation task while recording from the hippocampus and anterior cingulate cortex. They demonstrated that hyperglycemia impaired task performance and altered theta rhythm as well as increasing tau phosphorylation, which suggest there is potentially a direct link between chronic hyperglycemia and Alzheimer’s disease.

## Introduction

Alzheimer’s disease (AD) is a progressive neurodegenerative disorder characterized by escalating memory impairments and the presence of two core pathologies, amyloid beta (Aβ) plaques and hyperphosphorylated tau (ptau)^1–4^. Over the past 15 years, neuroinflammation has emerged as a third core pathology in AD^5–7^. Additional data have demonstrated that neuroinflammation promotes the production of Aβ and ptau^8,9^, demonstrating a role in the progression of the disorder^10^. While the exact cause of most AD cases (late-onset AD) is still unknown, a number of risk factors have been identified. These include genetic factors ApoE4^11^, age^12^, and other existing pathologies such as diabetes mellitus (DM), in particular, Type 2 DM (DM2). DM2 is characterized by insulin receptor insensitivity and hyperglycemia that eventually progresses to a more robust loss of insulin signaling^13^. Additional metabolic and vasculature changes are observed, as well as increased inflammatory signaling^14–16^. DM itself has been linked to cognitive and neurological problems^17^; however, little is understood about how or if DM2 affects mechanisms important for cognition.

The hippocampus is an area of vital importance for working memory function and is also one of the first areas in the brain affected by AD pathology^18,19^. DM and AD share multiple neuropathologies in the hippocampus, including impaired neurogenesis^20–22^, dendritic atrophy^23^, tau hyperphosphorylation, and increased neuroinflammation^10^. All these results were identified in the streptozotocin (STZ) model^10^. STZ is a diabetogenic drug that selectively damages insulin-producing pancreatic β-cells, leading to impaired insulin production^24^. High-dose STZ treatments lead to symptoms mimicking Type 1 diabetes, including severe insulin production impairments, massive weight loss, malaise behavior, and increased mortality^25,26^. Severe cognitive and learning deficits also occur, with impairments found in spatial memory^27^, working memory^28^, and hippocampal long-term potentiation (LTP)^29^. However, it is difficult to delineate the cognitive impairments from changes associated with illness and malaise behavior in this model. Murtishaw et al.^10^ employed an intermittent low-dose STZ model that mimics progressive and sustained hyperglycemia seen in late-stage DM2 known as “pancreatic exhaustion,” in an otherwise healthy animal. Importantly, in this model, learning and memory impairments were present, reiterating conclusions from previous studies that sustained hyperglycemia, and not sickness, induces cognitive impairments. Additionally, this model renders pathological changes in the brain consistent with observations in AD model systems, including increased tau phosphorylation and sustained immune response (chronic neuroinflammation). While impairments with hippocampal neurogenesis, LTP, and atrophy provide some insight into the mechanisms behind DM-linked cognitive impairments, they do not necessarily account for such large deficits^30^. Furthermore, the overlap of AD-related changes in the hyperglycemia model merits further investigations to determine whether changes in network-level function in the STZ model also mirror changes observed in AD-specific models.

We hypothesized that the learning and memory impairments previously observed in the low-dose STZ model were in part due to hippocampal network activity disruptions. During learning and memory tasks, hippocampal activity is dominated by the appearance of high-powered theta oscillations (6–13 Hz in rodents), which are integral for many of the mechanistic aspects thought to subserve hippocampal memory processes. Theta oscillations help to organize the flow of activity along the perforant path^31^, integrate entorhinal cortical input^32^, control neuroplasticity^33,34^, and are coupled with gamma oscillations to incorporate local neuronal activity with global hippocampus activity^35^. Perhaps equally important is theta rhythm’s role in coordinating interactions with the anterior cingulate cortex (ACC)^36–38^ and many other neural areas^39–41^. Interestingly, in various AD animal model systems, changes have been found in these very same theta-related hippocampal network functions^42–44^. However, hippocampal–cortical theta interactions have not previously been studied in a model of hyperglycemia and linking these interactions with hyperglycemia could provide a cognitive link between AD and DM.

To assess the effects of STZ-induced hyperglycemia in otherwise healthy animals on neural network activity, we recorded simultaneously from area CA1 in the hippocampus and ACC while rats performed a variable length delayed alternation task on a T-maze. We found that hyperglycemic animals had decreased theta/delta (TD) ratios in the hippocampus and, overall, had unique network activity in both areas, including elevated theta/gamma coupling. During the delay period, STZ animals had higher ACC–hippocampal theta coherence. On error trials, coherence was higher than for correct trials in STZ animals, but for controls, the opposite was true (i.e., more theta coherence leads to better performance). This effect was magnified on long delay trials, where STZ animals also had poor working memory performance. Consistent with previous work, we found increased levels of ptau in the hippocampus in STZ animals. Overall, we report significant changes in both hippocampal and ACC network activity and the interactions between these areas during working memory performance, similar to network disruptions observed in the early pathological stages of several different AD animal models.

## Results

### STZ-treated animals have high blood glucose

**Fig. 1:**
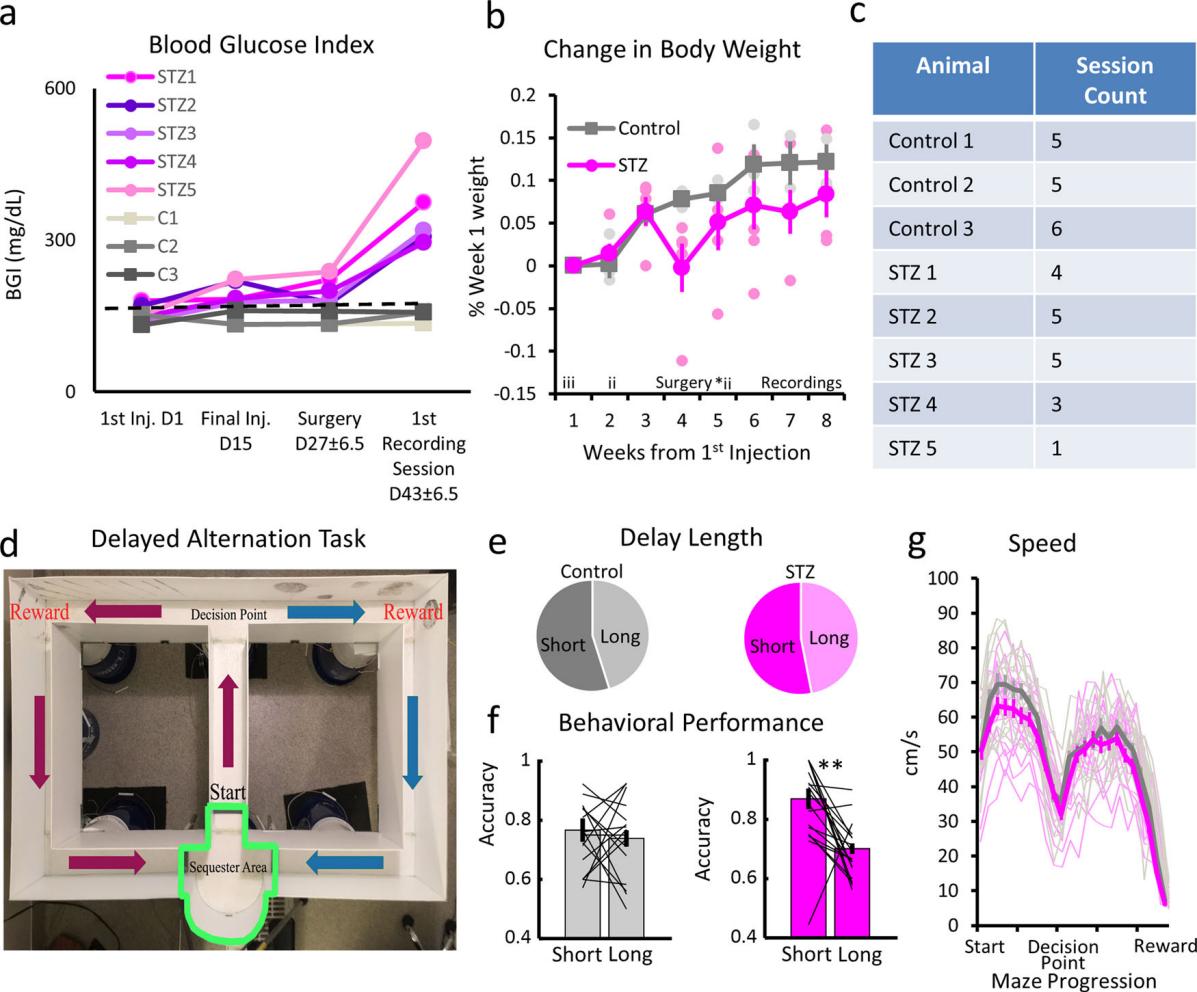
Metabolic and behavioral data. **a** Blood glucose values from all subjects. Blood glucose index values are shown on the *y*-axis and the date of testing is on the *x*-axis. **b** Group body weight change. All weights were normalized to week 1 values for each subject, which was the day of the final STZ or vehicle injections. i = injection day; *i = supplemental injection day. **c** Table of subjects and sessions. **d** Schematic of T-maze. **e** Distribution of trial delay lengths. **f** Accuracy by the total trial length between groups. Proportion of correct trials is on the *y*-axis, and total delay length is on the *x*-axis. **g** Mean running speed during trials. Running speed in centimeters per second is on the *y*-axis and position on the maze on the *x*-axis. **p < 0.01. Error bars = SEM.

Following the series of STZ injections, all animals in the experimental group exhibited a sustained fasting blood glucose reading >250 mg/dl (see Fig. 1a) and a mild decrease in body weight (see Fig. 1b), which normalized before recordings began. A threshold of ≥250 mg/dl was selected based on clinical criteria to establish a chronic hyperglycemia state consistent with DM^10^. The experimental group mean was 358.1 ± 36.7 mg/dl prior to the first recording session. A two-factor analysis of variance (ANOVA) revealed significant main effects for group (*F*(1,35) = 49.4; *p* = 1.2 × 10^−7^), day (*F*(3,35) = 13.05; *p* = 1.63 × 10^−5^), and a significant interaction (*F*(3,35) = 14.06; *p* = 8.9 × 10^−6^; Fig. 1a). These results align with our previously published work with this same protocol^10^.

We compared body weights over the course of injections and recordings with a two-factor ANOVA (Group × Weeks) and found significant main effects for group (*F*(1,61) = 6.93; *p* = 0.01) and weeks (*F*(7,61) = 7.44; *p* = 5.6 × 10^−6^), but notably no significant interaction (*F*(7,61) = 1.28; *p* = 0.28; Fig. 1b). While the increase in weight is expected over weeks, the group difference likely arose in the period immediately following surgery. The week following surgery (approximately the fourth week after the first injections), STZ animals lost considerable weight compared to controls; however, they quickly recovered, and no difference between groups was observed during recording sessions. It was likely that STZ animals recovered from surgery more slowly but quickly caught up to their control peers. All recording sessions that generated data analyzed in the present study were carried out 5.5 weeks or longer post-surgery when no significant weight differences were present.

### Behavioral impairments only appear during the longest delay trials

All animals performed a spatial delayed alternation task on a T-maze (see Fig. 1d). Animals were trained and reached criterion performance on the task before their first injection and prior to surgery (see “Methods”). On any given trial, an animal must navigate from the sequestered delay area around the maze via the return arms. Once animals returned to the delay area, they were sequestered for a random interval between 5 and 45 s (time spent sequestered). A total of 35 sessions were analyzed here: 19 sessions from 5 STZ animals and 16 sessions from 3 controls (see Fig. 1c). Trials were divided into short (<20 s) and long (>20 s) delay periods, depending on the time spent sequestered. This led to a relatively even distribution of trials, with slightly more short delay trials (Fig. 1e). We then compared the percent of correct trials by delay length between groups and found that STZ animal performance was strongly affected by delay length (Fig. 1f). A two-factor ANOVA (Group × Delay Length) found a significant main effect for Delay Length (*F*(1,66) = 9.14; *p* = 0.0036), but not Group (*F*(1,66) = 0.94; *p* = 0.33). Importantly, there was a significant interaction between Group and Delay Length (*F*(1,66) = 7.96; *p* = 0.0063) and follow-up tests showed the difference was isolated to the STZ group (Tukey’s; *p* < 0.001). These results show that STZ animals had a significantly larger drop off in accuracy for long delay trials compared with control animals. This is consistent with previous reports of memory problems with this same animal model^10^.

To ensure that group performance differences were not associated with general motor or sensory impairments, we examined the distance animals moved throughout the session and speed. We found the mean total distance traveled over all sessions was comparable *(F*(1,34) = 0.0027; *p* = 0.985). Since both theta and delta oscillations are linked with running, we compared running speeds during trials to assure that this behavioral factor did not confound our electrophysiological results. A two-factor ANOVA (Group × Maze Position) on running speeds from the sequester box to reward zone was performed, and while we did find that both main effects were significant (*p* < 0.01), notably we found no significant interaction between the two factors (*F*(23,792) = 0.619; *p* = 0.918; Fig. 1g). This indicates that, while overall speed between groups was different and speed varied over the maze, there was no section of the maze in which control and STZ animals ran at different speeds. As shown in the raw session traces in Fig. 1f, there is plenty of overlap between control (gray) and STZ (magenta) running speeds. No other behavioral differences were found between groups that might potentially explain any electrophysiological differences.

### Decreased hippocampal TD ratios in hyperglycemic animals

Local field potentials were recorded from the hippocampus and ACC of 8 animals (STZ = 5; control = 3). We included ACC recordings to analyze hippocampal–cortical theta interactions and to serve as a positive control for spectral analyses of the hippocampus, since hyperglycemia has been previously shown to strongly affect the hippocampus^10^. Recording implants targeted area 24 in the ACC^45^ and dorsal CA1 in the hippocampus. The location of the leads relative to the CA1 cell layers was inferred using methods from Mizuseki et al.^46^. We performed a two-way ANOVA on hippocampal sharp-wave ripple amplitude between groups and found no difference in STZ-treated or control animals (*F*(1,33) = 0.305; *p* = 0.584), confirming that hippocampal recording locations were comparable across subjects.

**Fig. 2:**
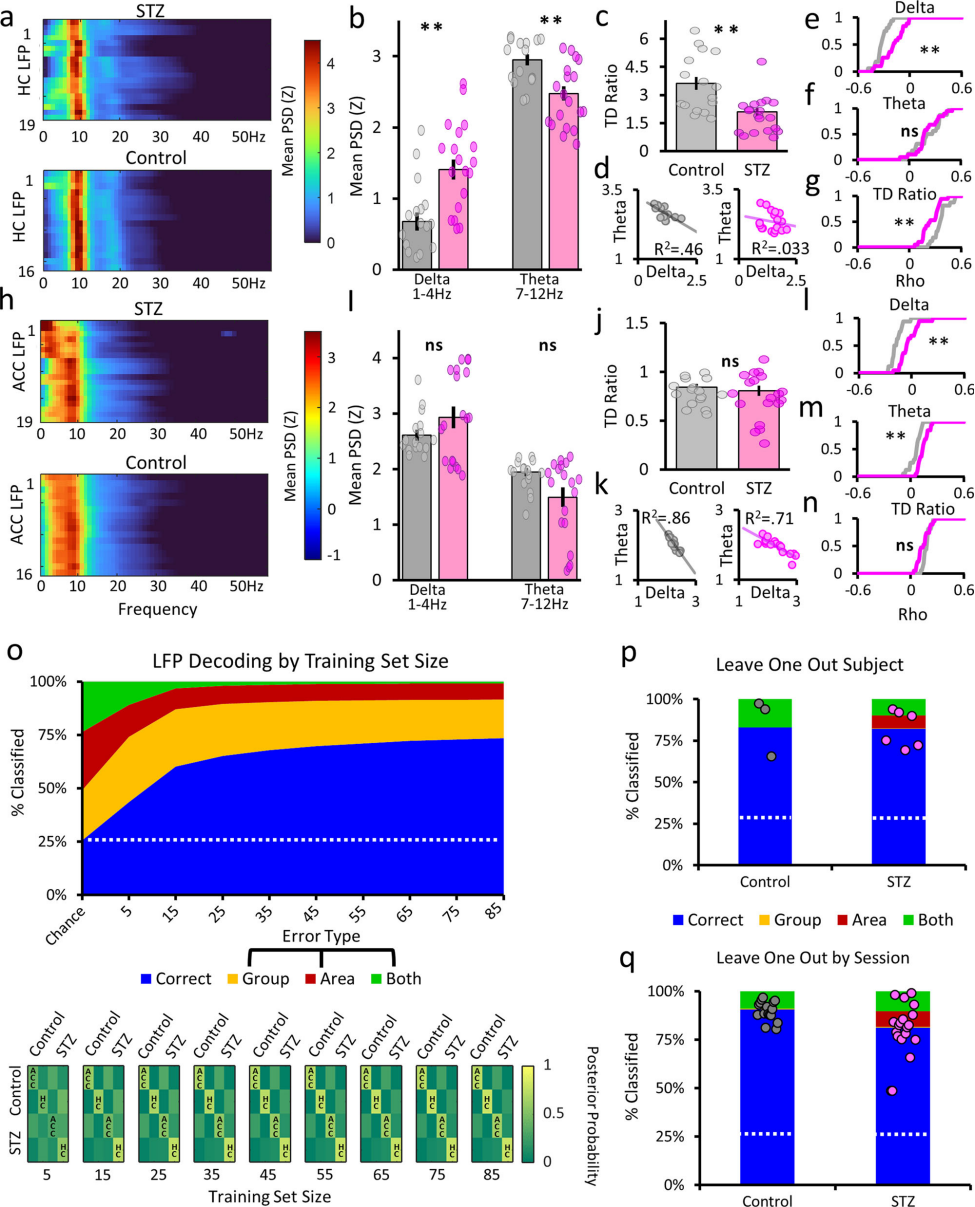
Spectral analyses of hippocampal and ACC local field potentials. **a**, **h** Power spectral densities (PSD) of each STZ (top) and control (bottom) group. Values are means from all leads from the entire recording session and have been normalized as a proportion of total spectral power. Sessions are on the *y*-axis and frequency is on the *x*-axis. **b**, **i** Mean power by frequency band. Mean normalized PSD power is on the *y*-axis and frequency bands are on the *x*-axis. Error bars show the standard error of the mean. Inset, mean spectral density over all leads per group by frequency (*x*-axis). **c**, **j** Theta/delta ratios. Mean theta power divided by delta power is on the *y*-axis. **d**, **k** Correlation between theta and delta power. Theta power is on the *y*-axes and delta power on the *x*-axes. Left, control sessions and right, STZ sessions. **e**–**g**, **l**–**m** Relationship between delta (**e**, **l**) and theta (**f**, **m**) power and theta/delta ratio (**g**, **n**) with running speed. Cumulative density of leads is shown on the *y*-axis and correlation values are on the *x*-axis. **h** ACC power spectral densities. Mean session long mean values for STZ (top) and control (bottom) animals. **o** Spectral phenotype analysis. Decoding accuracy by training set size. On the *y*-axis is percent accurately identified and the *x*-axis shows the number of LFP signals in each training set group. There were four groups: control ACC, control hippocampus, STZ ACC, STZ hippocampus. Colors show how leads were classified—blue for correct categorization and yellow, red, and green for the three types of incorrect classifications. The white dashed line shows the chance levels derived from 1000 bootstraps of randomly assigned group memberships. Bottom, confusion matrices by training set size. Colors depict the mean posterior probabilities for each category. Note the emergence of yellow on the diagonal as training set sizes increase. **p**, **q** Leave one out subject and session. On the *y*-axis is percent identified and the two groups are shown on the *x*-axis. Each dot (gray for control; magenta for STZ) shows an individual subject’s or sessions mean percent accurately identified. The white dotted line shows chance levels. In all plots, control values are in gray and STZ are shown in magenta. Dots indicate individual session values. **p < 0.01. Error bars = SEM.

Our initial set of analyses of hippocampus and ACC networks sought to characterize the global electrophysiological effects on slow frequency oscillations of chronic hyperglycemia. We first examined the hippocampal local field potential (LFP) power spectrum over the course of the full sessions (Fig. 2a), where we observed the most striking differences in the low frequency bands. For these analyses, each recording lead was spectrally decomposed and then values were averaged across all hippocampal leads, yielding a single value per session. Control animal CA1 leads were dominated by ~8 Hz theta activity, while hyperglycemic animals often had very strong delta (1–4 Hz). We found significant delta power in hyperglycemic animals (*F*(1,33) = 14.14; *p* = 6.6 × 10^−4^) across all CA1 recordings and subjects (Fig. 2b). Also, we found significantly lower theta power in STZ animals (*F*(1,33) = 13.44; *p* = 8.57 × 10^−^^4^). These differences show that CA1 delta and theta oscillations were affected by chronic hyperglycemia during spatial working memory performance.

The hippocampus typically alternates between active processing theta states and offline delta states^47,48^. Thus, throughout the delayed alternation task, processing states are regulated by the task structure, as animals alternate from periods of running to stationary periods during reward consumption and the delay period. Typically, theta power dominates during the running periods and delta appears more prominently in the absence of movement. Since our previous analysis found group differences in delta and theta power, we next investigated whether the interplay between these two oscillations was disrupted. To quantify this relationship, we calculated TD ratios, a prominent measure used to find alterations associated with AD pathology in both humans and animal models^49^. We calculated TD ratios for the entire sessions, which captured the strength of the changes in these two frequency bands. By averaging power over the entire session, we wanted to “smooth” out any transient behavioral influences that could mask more enduring changes in oscillatory balance. We found that TD ratios were lower in hyperglycemic animals (*F*(1,33) = 20.03; *p* = 4.66 × 10^−4^; Fig. 2c). These results demonstrate that the interplay between these bands was altered at the recording sites over the full length of the session, similar to patterns associated with AD pathology.

TD ratio differences indicate a fundamental change in the coordination of these behaviorally relevant network states, but this analysis does not address what factors lead to the decreases. Smaller TD ratios could arise if theta power was lower but delta power was unchanged, or if delta was higher but theta was unchanged, or if the relationship between the two was altered through some combination of these factors. In turn, when we examined how theta and delta power related during a given session, we found a strong negative correlation in controls (*R* = −0.683; *p* = 2.97 × 10^−7^); however, this relationship was not present in hyperglycemic animals (*R* = −0.185; *p* = 0.45; Fig. 2d). These results show that the fundamental balance between these two behaviorally relevant oscillations was altered by chronic hyperglycemia, but understanding what factors contributed to this imbalance can be difficult. When we examined behavior over the whole session, animals in both groups performed a similar number of trials, ran at similar speeds, and had similar distributions of trial lengths, and thus, it is reasonable to conclude that these differences were not due to changes in gross behavior. However, since both theta and delta are strongly affected by locomotion and running speed^50^, it remains possible that changes in TD ratio might be linked to more discrete behavioral changes.

Our next set of analyses examined whether the moment-to-moment coupling between delta and theta oscillations with running speed was altered in STZ animals. In control animals, delta power was negatively correlated with running speed, as expected; however, in STZ animals this relationship was weaker (*Χ*^2^(1,33) = 9.89; *p* = 0.0017; Fig. 2d). As can be seen in Fig. 2e, hyperglycemic animals’ cumulative distribution of delta/running speed correlations is shifted toward zero. For theta oscillations, we did not find a similar effect. (*Χ*^2^(1,33) = 0.246; *p* = 0.619; Fig. 2f), as both groups had equally strong positive correlations between theta and running speed. We next compared TD ratios with running speed over the course of the session and again found that, across all sessions, STZ animal’s hippocampal networks were less coupled with running speed (Fig. 2g). These analyses lead to multiple conclusions. First, it appears that behavioral differences do not account for the observed effects in delta power and TD ratio since STZ animal’s delta, theta, and TD ratios were differentially affected by ongoing behavior. Clearly, STZ treatments and factors related to the resultant long-lasting hyperglycemia are fundamentally changing delta oscillations in hippocampal networks. In STZ animals, the antagonistic relationship between theta and delta oscillations was less prominent throughout the task session, and the interplay between these oscillations (i.e., TD ratio) was less influenced by locomotor behavior over trials during sessions. Additionally, these results suggest that higher delta power during running was affecting whole-session TD ratios, as opposed to delta during stationary periods. This can be seen in the weaker negative relationship between STZ animals’ delta power and TD ratios with running, while theta correlations remained like controls. Taken together, these analyses suggest that differences in delta power and delta’s relationship with ongoing behavioral output were primarily responsible for the changes in TD ratio, though theta changes might have also contributed.

### ACC delta and theta power more coupled with running

Oscillatory dynamics in the ACC are generally more diverse than in the hippocampus. While there is prominent theta activity, there is also considerable delta power present during task performance^51^. In STZ animals, there were many instances where we found peaks in the delta range or two clear peaks, one in delta and another in theta (Fig. 2h). When we examined individual frequency band mean power between groups, we did not find significant changes in delta (*F*(1, 33) = 1.95; *p* = 0.172) and found a slight but not significant (corrected for multiple comparisons) difference in theta power (*F*(1,33) = 4.977; *p* = 0.0326; Fig. 2i). We also found no difference in TD ratios in ACC (*F*(1,33) = 0.378; *p* = 0.542; Fig. 2j). Overall, we found much smaller effects of chronic hyperglycemia in ACC oscillatory power, which can be readily seen in the correlations between whole session delta and theta power (Controls: *R* = −0.93; *p* = 2.97 × 10^−7^; STZ: *R* = −0.84; *p* = 5.87 × 10^−6^; Fig. 2k).

We next examined the relationship between theta and delta power in the ACC, by analyzing the power in each band and how it related to the animal’s running speed. Relative to controls, we found that a much larger percentage of STZ ACC leads had delta power that was less negatively correlated with running speed (*Χ*^2^(1,33) = 14.754; *p* = 1.22 × 10^−4^; Fig. 2l). In STZ animals, significantly more ACC leads showed a strong positive correlation between theta and running speed (*Χ*^2^(1,33) = 12.302; *p* = 4.84 × 10^−4^; Fig. 2m). Lastly, we found no noticeable differences in how ACC TD ratios related to running speed in the STZ and control groups (*Χ*^2^(1,33) = 2.965; *p* = 0.085; Fig. 2n). Thus, chronic hyperglycemia increased the coupling between both theta and delta oscillatory power and locomotor behavior, but it did not affect how the relative strength of these frequency bands related to running speed. Unlike in the hippocampus of STZ animals, the lack of differences in TD ratio or its relation to running suggest that, in ACC, there was a general increase in lower frequency oscillatory linkage with running. Thus, higher delta power and delta coupling with running were offset by higher theta power and theta running coupling, leading to unchanged TD ratios. All together, these results reveal that cortical oscillatory activity was also affected by STZ in multiple frequency bands, though these effects were more nuanced than those observed in the hippocampus.

### Identifying a unique electrophysiological phenotype due to hyperglycemia

After finding changes in multiple frequency bands (notably delta and theta) in both the ACC and hippocampus of STZ animals, we next sought to determine how widespread these effects were throughout all our subjects and recording leads. We hypothesized that, if hyperglycemia fundamentally altered network activity in these areas, this should be relatively consistent across animals, recording sites within areas, and sessions. Thus, there should be an identifiable spectral pattern that can distinguish ACC and hippocampal leads in controls vs. STZ animals. Thus, we had 4 categories and we used the normalized spectral power between 1 and 100 Hz for classification. We classified with support vector machines (SVMs) and first examined accuracy for different sized training sets. For each iteration, we randomly selected the same number of leads for each category to use to train the classifiers, and the remaining leads were then decoded. We repeated this process 1000 times for each training set size in multiples of 10 from 5 to 85 leads. Figure 2o shows the results for the different sized training sets and shuffled data with 85 leads. We noted whether it was accurately classified or misclassified for each test lead, by group (STZ vs. control), area (ACC vs. hippocampus), or both group and area. We found an equal likelihood for each of the 4 outcomes for our shuffled data, or that chance was indeed 25%. With as few as 5 leads per training set, we found that we could get above chance classification accuracy. With 15 leads, accuracy was >50% and started to plateau at 45 leads per training set. Maximum accuracy was found with 85 leads per training set at 73.4%. Furthermore, the posterior probabilities increased as larger training sets were used (Fig. 2o). This analysis shows that STZ treatments lead to a unique oscillatory profile in the hippocampus and ACC.

To confirm that STZ animals had an identifiable electrophysiological phenotype, we tried to classify an animal’s leads based upon other animals. We ran a series of leave one subject SVM analyses with training sets equally composed of control and STZ sessions from other subjects. We then attempted to classify the leads from our “left out” subject. This process was repeated 100 times per subject. We found that we were able to decode both group and area with a high degree of accuracy (control = 86.7% and STZ = 82%; Fig. 2p). This finding clearly shows that animals treated with STZ had consistent changes in both hippocampus and ACC oscillations across animals.

As a control, we next sought to determine whether we could accurately classify leads from one session based on training sets from other sessions. We ran another SVM analysis, but this time using all but one session as training sets and then attempted to classify the remaining session. We randomly selected an equal number of control and STZ sessions and repeated this 100 times for each of the “left out” sessions. We again found very high classification accuracy (control = 90.5% and STZ = 81%; Fig. 2q). Together these classification analyses confirm our hypothesis that chronic hyperglycemia leads to a unique oscillatory phenotype in both the hippocampus and ACC during the performance of a spatial delayed alternation task.

### Elevated cross-frequency coupling in hippocampus and ACC

**Fig. 3:**
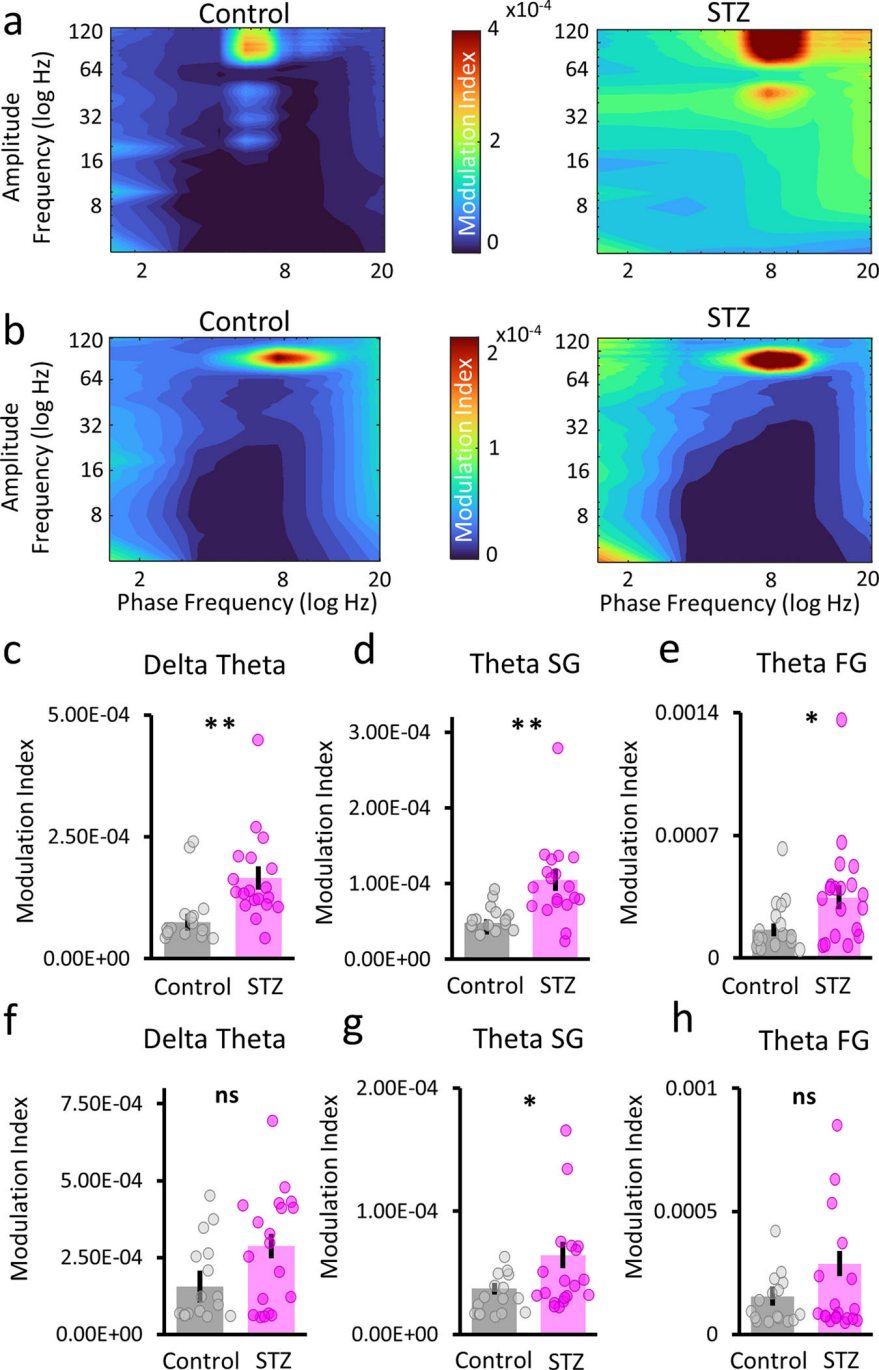
Hypersynchrony in STZ subjects. **a**, **b** Mean hippocampal (**a**) and ACC (**b**) comodulograms for the control (left) and STZ (right) groups. Amplitude frequency is on the *y*-axis and phase frequency is on the *x*-axis. Modulation index value is on the *z*-axis and the scales in **a**, **b** apply to both control and STZ plots. **c**–**e** Hippocampal LFP modulation index comparisons. For all comparisons, the phase frequency is listed first and the amplitude frequency second. Modulation index values are on the *y*-axes. **f**–**h** ACC LFP modulation index comparisons. Same as described above for hippocampus. In all plots, control values are in gray and STZ are shown in magenta. **p < 0.01; *p < 0.05. Error bars = SEM.

Our previous analysis had shown that STZ animals have considerable changes in multiple oscillatory frequency bands in both hippocampus and ACC; we next investigated the possibility that the coordination between these different oscillations may also be impacted by hyperglycemia. We first analyzed cross-frequency phase–amplitude coupling (PAC) and found that overall STZ animals’ networks were more strongly coupled relative to control animals. As shown in the comodulograms in Fig. 3a, b, hypersynchrony was widespread across frequency bands in both hippocampus and ACC. When we analyzed cross-frequency coupling in specific frequency bands, we found unique patterns in both hippocampus and ACC. In the hippocampus, we found that delta–theta (*Χ*^2^(1,33) = 11.64; *p* = 6.48 × 10^−4^; Fig. 3c), theta–slow gamma (*Χ*^2^(1,33) = 11.54; *p* = 2.84 × 10^−^^8^; Fig. 3d), and theta–fast gamma PAC (*Χ*^2^(1,33) = 6.17; *p* = 0.013; Fig. 3e) were all elevated in STZ animals. In the ACC, we found significant increases in theta–slow gamma PAC (*Χ*^2^(1,33) = 5.07; *p* = 0.0243; Fig. 3g); however, unlike in the hippocampus, there were no differences in delta–theta (*Χ*^2^(1,33) = 1.583; *p* = 0.208; Fig. 3f) or theta–fast gamma PAC in STZ animals (*Χ*^2^(1,33) = 0.088; *p* = 0.77; Fig. 3h). Overall, we found evidence of hypersynchrony in STZ animals in both the hippocampus and ACC that manifested as in different overcoupled frequency bands, but most notably between theta and slow gamma.

### Hippocampus and ACC oscillatory changes during the delay period

After establishing that both hippocampus and ACC network activity was altered in STZ animals, we next wanted to assess whether these changes could account for the behavioral impairments found in STZ animals. Specifically, we were interested in activity during the delay period, where behavior was limited for all animals. Since our previous analyses investigated oscillatory changes over the entire session, it is possible that these effects might dominate during other times and be absent during the critical delay periods. Previous research has shown that delay period activity in both the hippocampus and ACC is of singular importance for successful spatial working memory performance^52^.

**Fig. 4:**
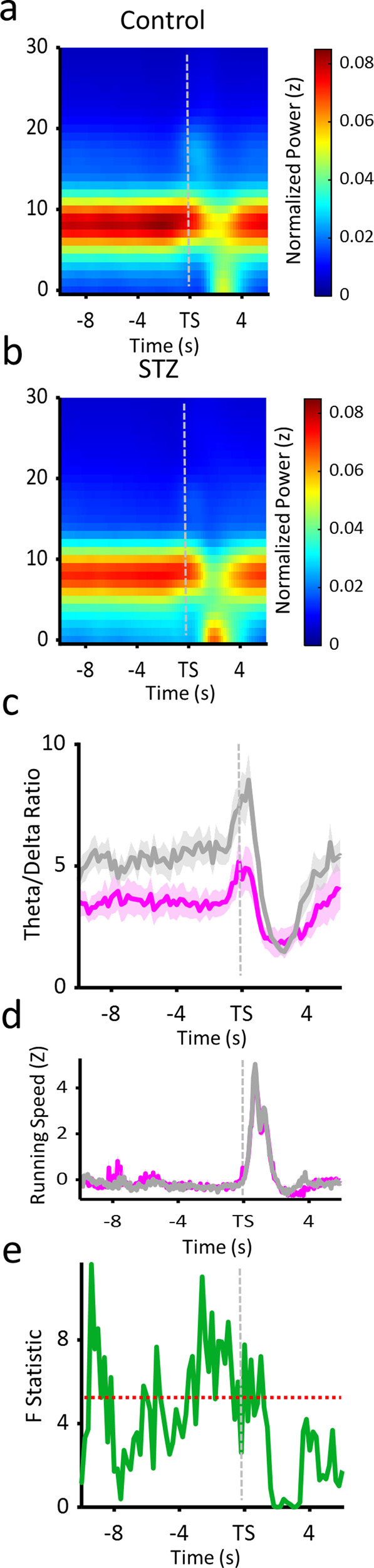
Theta delta ratios during the end of the delay period. **a**, **b** Mean peri-event spectrograms for the hippocampus of control (**a**) and STZ (**b**) groups. Frequency is shown on the *y*-axis and time relative to trial start (TS) is on the *x*-axis. **c** Mean peri-event theta delta ratios for the hippocampus. TD ratio values on the *y*-axis and time relative to trial start is on the *x*-axis. Gray lines show control group value and magenta show STZ. Shaded error bars show standard error of the mean. **d** Mean running speed. Normalized running speed values are shown for the control (gray) and STZ (magenta) groups over the same time period as plots **c**, **d**. **e** Running *F* tests for TD ratios for the hippocampus. TD ratios from all trials were compared at each time step (500 ms) between the two groups with a single-factor ANOVA. *F* value is on the *y*-axis and time relative to trial start is on the *x*-axis. The red dotted lines show the level for significance at *p* < 0.05; Bonferroni corrected. Error bars = SEM.

We first examined oscillatory power changes during the interval at the end of the delay period. As illustrated in Fig. 4a, in the control animals’ hippocampus, spectral power during the end of the delay period was dominated by strong theta activity, which became even stronger once the delay ended and the animals started their trial run. For STZ animals, hippocampus power also showed strong theta activity, but this was accompanied by relatively high-powered delta activity that also increased when animals started trials (Fig. 4b). We calculated TD ratios for the end of the delay period and found that control TD ratios were higher than STZ animals throughout the delay (Fig. 4c). This finding confirmed that the overall session differences in TD ratio, shown above, were indeed present during this crucial trial epoch. At the very end of the delay, control animals’ TD ratios increased and peaked just after the start of the trial. While STZ animals also showed an end of the delay increase, it was not nearly as strong as what we found in controls. Notably, these effects seem to be somewhat independent from running speed-related TD ratio changes. As can be seen in Fig. 4d, running speed is comparable for each group throughout the delay period and trial run, while larger TD ratios for control animals are present during the delay and then ramp up leading up to the initiation of running. This effect can be seen clearly in the *F*-values comparing TD ratios between groups in Fig. 4e (df(1,33)). While many time points during the delay were above significance levels (dotted red line), the prevalence of significant differences ramped up toward the end of the delay and into the start of the trial run. These results show that the altered hippocampal oscillatory balance between theta and delta was present during the delay period and worsened as the end of the delay approached.

### Delay period hippocampus–ACC coherence changes correlate with trial accuracy

We next examined whether oscillatory changes due to hyperglycemia could account for task accuracy. ACC–hippocampus theta coherence is known to be of great importance for proper working memory function. In spatial working memory tasks alone, ACC–hippocampus theta interactions are linked with accuracy at multiple time points throughout the task: during the delay period^53^, the trial run^54^, at the decision point^38,55^, and during the initial learning^56^. This work has consistently revealed a decrease in theta-based interactions during error trials compared with correct trials, suggesting that these interactions are necessary for accurate performance. Since we have found that STZ animals have changes in hippocampal TD ratios during the delay periods and evidence of theta band hypersynchrony within the ACC and hippocampus, what changes would we find in ACC–hippocampal theta coherence, and could such changes provide insight into why STZ animals performed worse on long delay trials? We examined ACC–hippocampus coherence during the end of the delay period up until the decision point in the maze.

**Fig. 5:**
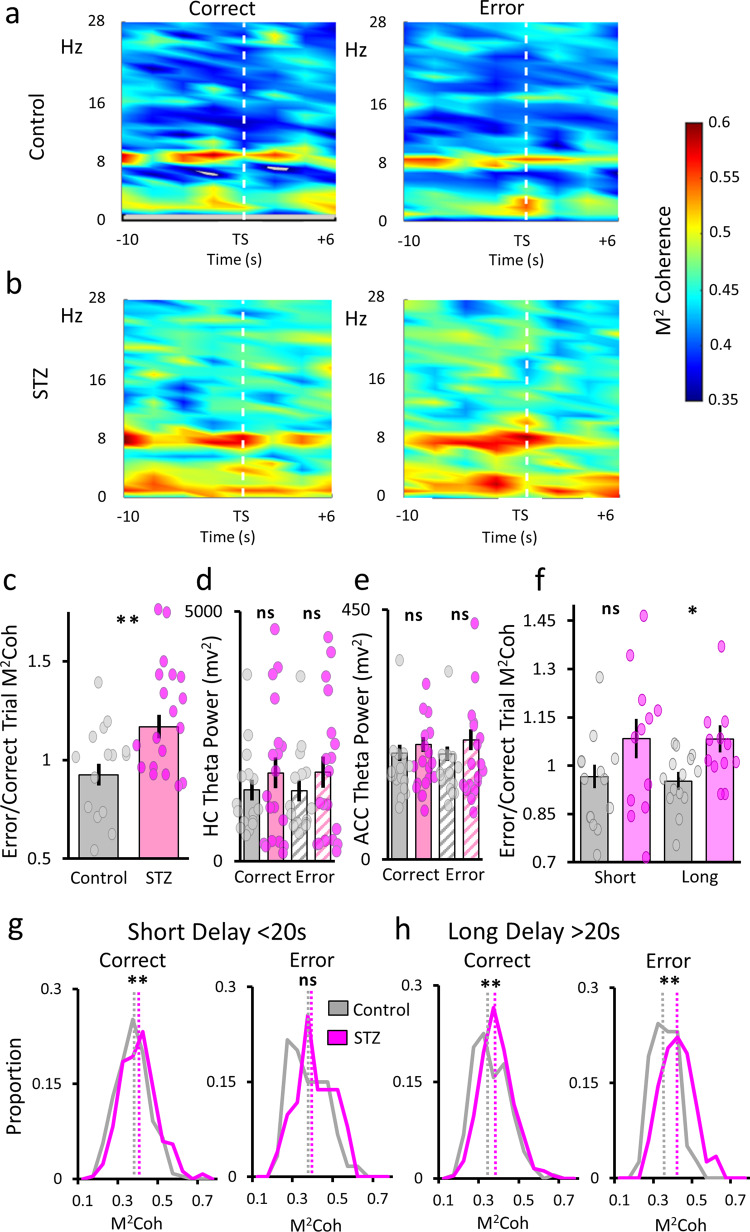
Coherence during the delay period for correct and error trials. **a**, **b** Peri-event ACC–hippocampus cohereograms for correct and error trials for the control (**a**) and STZ (**b**) groups. Frequency is shown on the *y*-axis and time relative to trial start (TS) is on the *x*-axis. The z-axis legend on the far right applies to all plots. **c** Theta coherence trial outcome difference scores. Mean error trial coherence divided by mean correct trial coherence per session. **d**, **e** Theta band power in the hippocampal and ACC. Spectral power is on the *y*-axis and group and trial outcome on the *x*-axis. Correct trials: solid bars; error trials: striped bars. **f** Coherence difference scores by delay length. **g**, **h** Distribution of theta coherence values for short (**g**) and long (**h**) delay trials. Control and STZ groups on correct (left) and error (right) trials. Magnitude squared coherence is on the *x*-axis and proportion of trials is on the *y*-axis. Dotted lines show the median coherence value. In all plots, control values are in gray and STZ are shown in magenta. **p < 0.01; *p < 0.05. Error bars = SEM.

As can be seen in the mean cohereograms in Fig. 5a, b, there is a clear band of increased coherence in the theta range. In control animals, theta coherence values appear stronger during the end of the delay period and the first few seconds of the trial run on correct trials. STZ animals appear to have the opposite pattern, where theta coherence is decreased on correct trials. To examine these possibilities, we calculated mean ACC–hippocampal theta coherence values over the end of the delay period and trial run for both correct and error trials. We then calculated a simple difference score by dividing mean error coherence by mean correct coherence per session. We compared these values with a single-factor ANOVA and found that STZ animals had a larger error/correct difference scores (*F*(1,33) = 8.54; *p* = 0.0062; Fig. 5c). The control group mean difference score was <1, which is consistent with previous reports. However, hyperglycemic animals showed the opposite pattern, higher ACC–hippocampal coherence values during delay periods on error trials. Since magnitude squared coherence can be influenced by oscillatory power^57^, we compared the raw power of theta oscillations in both areas for correct and error trials. We performed two-factor ANOVAs with group and trial outcome as variables. We found no significant differences in theta power in either hippocampal (group: *F*(1,66) = 1.68; p = 0.199; trial outcome (*F*(1,66) = 0.000034; *p* = 0.99) or ACC (group: *F*(1,66) = 1.74; *p* = 0.192; trial outcome (*F*(1,66) = 0.043; *p* = 0.94). These results suggest that the differences we found in theta coherence between groups cannot be explained by differences in theta power.

Our next analysis sought to understand how theta coherence changes were related to working memory performance. For all our previous analyses, we grouped trials together and looked at mean values from all sessions, but to examine delay length and trial accuracy this was not possible. When we separated trials by delay and outcome (i.e., short delay correct, short delay error…), there were some sessions (mostly STZ) with very few trials in one of the four categories (mostly long delay correct or short delay error). We restricted this analysis to sessions with at least 3 trials in each category, leaving us with 12 of the 19 STZ sessions and 14 of the 16 control sessions. Since this left only a subset of available sessions and of the total trials, we employed two different approaches to understand how delay length and performance affected ACC theta coherence. First, we analyzed just the limited set of sessions with enough of each trial type and next we examined all trials together across animals and sessions. In both cases, we averaged coherence values within the session, so there was one value for each trial, and we found similar results.

We first compared the whole session averaged error/correct trial coherence difference scores for short and long delay trials. We found no significant difference on short delay trials (*Χ*^2^ = 2.54; *p* = 0.11), but on long delay trials STZ values were larger than controls (*Χ*^2^ = 5.59; *p* = 0.018; Fig. 5f). Thus, in hyperglycemic animals only on long delay trials were coherence difference scores higher, indicating that correct performance was related to lower coherence values relative to error trials and control animals displayed the opposite pattern.

The above analysis was limited by nature since not all sessions and trials were included, so to examine the full scope of this effect we examined the distribution of ACC–hippocampal delay period coherence values for all trials from all sessions and compared the two groups directly. The true nature of these changes is apparent in the distributions of theta coherence values for each trial outcome (Fig. 5g, h). For short delay trials, the distributions are similar between groups on error trials, though there is a slight skew to the right for STZ animals. During the long delay trials, however, theta distributions are highly divergent between groups for both correct and error trials. For the control group, error trial distributions are skewed to the left, indicating more low coherence values; however, the STZ group data are skewed to the right due to higher coherence values on error trials. Statistical significance tests largely backed up these observations. We found significant differences in the distributions of short delay correct trials (*Χ*^2^(1,449) = 10.83; *p* = 9.98 × 10^−4^; Fig. 5g), with the median STZ value being greater than the control value; however, there was no difference between groups for short delay error trials (*Χ*^2^(1,109) = 3.01; *p* = 0.083; Fig. 5g). During long delay trials, we found significant group differences for both correct (*Χ*^2^(1,482) = 11.812; *p* = 5.89 × 10^−4^; Fig. 5h) and error trials (*Χ*^2^(1,193) = 24.964 *p* = 5.84 × 10^−7^; Fig. 5h). This examination of the full range of trials from all animals shows the extent of hyperglycemia’s effects on ACC–hippocampal theta coherence during working memory delay periods.

The changes indicate two distinct types of wholesale changes in communication between the ACC and CA1; for healthy animals, a loss of synchrony during the long delay periods was more likely to lead to errors, but for STZ animals, lower coherence values lead to correct performance. This finding indicates that such high baseline levels of ACC–CA1 coherence in STZ animals were not optimal for the task’s working memory demands leading to error commission. Thus, the dynamics relating ACC–hippocampus synchrony with working memory were altered in the STZ animals. The hypersynchrony was so strong that a degree of desynchronization occurred on correct trials, which is opposite to what is found in healthy brains.

### Increased phosphorylation of tau in hippocampus and frontal cortex

**Fig. 6:**
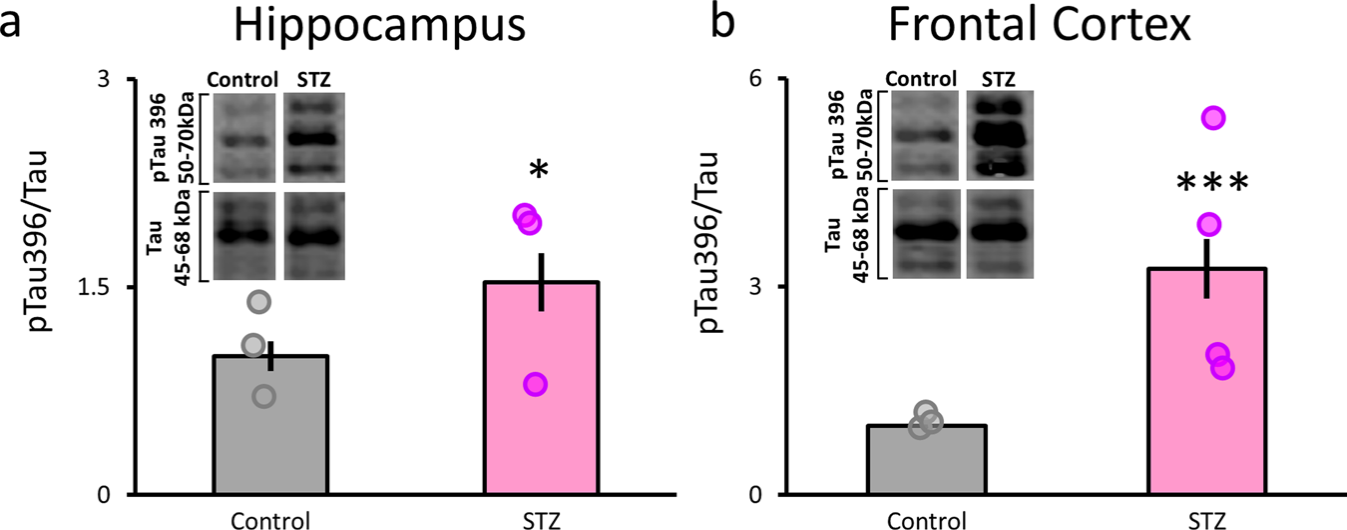
STZ leads to increased tau phosphorylation in frontal cortex (including ACC) and hippocampus. **a**, **b** Tau levels at Ser396 were significantly elevated in the hippocampus (**a**) and frontal cortex (**b**). Proportions of pTau396 normalized to total tau are on the *y*-axes. Dots show individual data points (see “Methods”). Saline control values are in gray and STZ in magenta. Representative western blot images are shown in the insets. *p < 0.05; ***p < 0.001. Error bars = SEM.

In previous reports STZ-induced hyperglycemia led to significantly increased phosphorylation of tau (ptau) proteins consistent with other animal models of AD^10^, providing a linkage to the increased risk DM confers to developing AD. This finding is critically relevant since tau pathology is the constituent of neurofibrillary tangles^58^ and has been linked with impaired network activity and cognitive dysfunction^59^. We found a significant increase in phosphorylation of the tau Ser396 epitope in hippocampus (*F*(1,20) = 5.09; *p* < 0.05; Fig. 6a and Supplementary Figs. 1–3) and frontal cortex, including ACC (*F*(1,20) = 20.02; *p* < 0.001; Fig. 6b). These results replicate our previous findings and demonstrate that the staggered and low-dose STZ protocol reliably leads to increased ptau levels in both hippocampus and cortex.

## Discussion

In the current study, we found that animals administered a low-dose STZ protocol, that induced prolonged hyperglycemia in otherwise healthy animals, had unique alterations in hippocampal and cortical network activity. We report three main findings: (1) hyperglycemic animals exhibited identifiable patterns of network changes in both ACC and hippocampus; (2) hypersynchrony within and between areas was observed in the hyperglycemic animals as compared to controls; (3) during delay periods prior to error trials, cortico-hippocampal communication moved in opposite directions for control and hyperglycemic animals. These data are novel since the impact of sustained hyperglycemia on overall hippocampal and cortical network function has not been well characterized in vivo. In addition, because hyperglycemia is a core feature of DM2, which confers increased risk for developing AD, the changes observed in the present study have implications in AD pathogenesis. Based on the links between DM and AD and the paucity of existing comparable studies in DM models, these results are discussed in relation to network changes related to AD pathology.

We found that STZ animals had increased delta oscillatory power in the hippocampus, which appeared to arise at the expense of power in the theta band; thus, the functional relationship between delta and theta was altered. In healthy animals, typically there is a negative relationship between delta and theta driven by changes in behavior, as hippocampal field potentials vacillate between periods of high-powered theta during movement and then strong delta power when the animal is still. In hyperglycemic animals, both delta and theta oscillations were decoupled from locomotor activity compared with controls, suggesting not only that the ebb and flow of delayed alternation behavior (i.e., trial runs and delay periods) did not coincide with appropriate balances of these oscillations, but also that the balance between the oscillations, in general, was disturbed. Since many cognitive processes related to spatial working memory rely on theta activity in both the ACC and hippocampus, any alterations in how theta relates to behavior could have profound effects. Indeed, transection of the fornix, which significantly decreases hippocampal theta power^60,61^, leads to spatial working memory deficits comparable to full hippocampal lesions^62^. While the current results revealed more mild decreases in hippocampal theta power, these differences resulting from a sustained hyperglycemic state are substantial. These findings also overlap with similar effect sizes observed in multiple animal models of AD^44,63,64^. Even slight reductions in theta power can lead to learning impairments and decreased spatial information in hippocampal cells^65^. Alternatively, studies have shown that increased delta power may also contribute to working memory performance. In fact, the stimulation of delta to the thalamic nucleus reuniens, an indirect ACC–hippocampal pathway, leads to elevated delta in the hippocampus and spatial working memory impairments^66,67^. Thus, this imbalance may be attributed to both higher delta power and lower theta power. Furthermore, we also found that the relationships between theta and delta with running speed were altered in the STZ group. This finding suggests that the overall dynamics of hippocampal network activity were changed in subtle ways. There is evidence that glutamatergic septal inputs control the coupling of theta oscillations with running speed^68^, but it is unknown how, or if, these projections are affected by STZ treatments and the resulting chronic hyperglycemic state. Additionally, no known mechanisms could explain why delta oscillations become less negatively correlated with running speed. However, given that delta and theta oscillations are generally orthogonal in the healthy hippocampus^47^, it is likely that any alterations to this antagonistic relationship could impact hippocampal function.

Using a machine learning-based group decoding approach, we revealed that, overall, STZ animals had unique LFP signals in both ACC and hippocampus. This analytical approach allowed us to identify an electrophysiological phenotype in the ACC and hippocampus of STZ animals. To our knowledge, this is the first time that LFP spectral analyses have been used to accurately identify treatment groups and recording location of other LFPs. In the present study, this novel analysis strongly supported our conclusion that chronic hyperglycemia fundamentally alters both hippocampus and ACC network activity. The sensitivity of this approach is quite notable, as evidenced by the ability to separate ACC control and STZ networks, even though traditional statistical approaches did not find any significant group differences in the dominant oscillatory power bands (delta and theta). This analytical approach could be applied to a vast range of translational studies and could also potentially serve as a diagnostic measure in human patients^69–71^.

In the STZ group, we found increases in cross-frequency coupling in both the hippocampus and ACC between multiple frequency bands. In the hippocampus, there was an increase in delta–theta coupling, though these oscillations are generally not linked in healthy subjects; however, there is also no established relationship between delta–theta coupling and memory. Indeed, ordinarily in the hippocampus delta and theta states appear in opposition to each other, as discussed above, so the idea of delta amplitude modulating theta phase is difficult to reconcile with the current understanding of normal hippocampus functioning. Though, in AD patients, reports have found that delta–theta coupling is increased^72–74^. Indeed, Ranasingeh et al.^75^ recently found that the accumulation of tau and Aβ peptides correlated with increased delta–theta synchrony in AD patients, showing that these frequency-specific interactions are likely affected by AD pathology. Given that we found increased tau in the hippocampus and ACC in STZ animals, we may have detected a similar effect in this hyperglycemia model.

The relationship between theta and gamma oscillations is better understood than delta–theta, at least in the hippocampus, but it is more complex. The activity of both fast and slow gamma in CA1 originates via different inputs. Fast gamma is driven by incoming entorhinal cortex input^76,77^, while slow gamma is influenced by CA3 input to CA1^77,78^. It is thought that, during active behavior, CA1 is switching back and forth between entorhinal cortex and CA3 afferent drive corresponding with changes between encoding and retrieval of memory information^79^. In turn, there is evidence that theta–fast gamma coupling is linked with improved learning^35^ and theta–slow gamma coupling is linked with memory retrieval^80^. Perhaps, the increases we found in both comparisons could counteract each other; however, it is more plausible that these alterations are hampering the separation of the different phases of memory, leading to inefficient and unreliable memories. Interestingly, decreases in CA1 theta–gamma coupling have been found in multiple amyloidosis models^81,82^, but the role of accumulated tau on theta–gamma coupling is less clear. Tanninen et al.^83^ found that injections of ptau fragments into the entorhinal cortex lead to decreased learning-related theta–slow gamma coupling in the hippocampus but no effects on theta–fast gamma coupling. In the current results, we found increased tau levels in the hippocampus and increased theta–fast gamma coupling in the hippocampus. It is not difficult to imagine that the effects of accumulated tau within the hippocampus are likely to be different than when tau is in the entorhinal cortex only, but certainly, more work is needed to unravel these effects. Another unaccounted result is the increase we found in theta–slow gamma coupling in the hippocampus, as most studies in AD models have shown that increased tau leads to decreases in theta–slow gamma cross-frequency coupling^84,85^. It is possible that the data collected here represent an intermediate state between what occurs in controls prior to more substantial disruption by AD pathology. Undoubtedly, more work is needed to understand the complicated relationship between ptau and cross-frequency coupling.

Additionally, we found highly elevated inter-area synchrony between the ACC and hippocampus in the theta band. Synchronization of theta rhythms between the ACC and hippocampus has been strongly linked with working memory performance^38,54,55^, and any changes to these interactions are likely to impact cognitive function. The mechanisms underlying these effects are less clear, but there have been widespread reports of hypersynchrony in a range of animal AD models^86^ and human patients^87,88^, including seizures along with the high comorbidity of epilepsy and AD^89–91^. There are also possible links to changes in excitatory–inhibitory (E/I) balance, which are thought to also underlie epilepsy, and it has recently been proposed that changes in the E/I balance occur as AD pathology and cognitive symptomology progress^92^. It will take more experimentation to better understand the mechanisms behind inter-area hypersynchrony and how AD-related pathology affects this phenomenon.

The cognitive challenges that hypersynchrony creates are robust. Since first suggested by Gray^93^, the neural synchrony hypothesis has been widely supported by extensive neural data in a range of species^94^. In the current experiments, our control group had decreased ACC–hippocampus coherence during error trials, indicating a lack of communication between these areas and replicating the purest essence of neural synchrony hypothesis (i.e., synchrony supports information transfer enabling cognitive success). Similar theta range effects have been found in spatial working memory and long-term memory tasks before in rodents^53,54,95^, macaques^96^, and in humans^97,98^. Thus, it is quite remarkable that we found that in STZ animals coherence between the ACC and hippocampus was decreased on correct trials. This suggests that coherence between these areas might have a U-shaped function, such that too little or too much coherence can hinder performance. To our knowledge, this is the first report of desynchronization between the ACC and hippocampus being linked with improved working memory performance in any animal model. Given that multiple reports have recently showed that patients with mild cognitive impairment and early AD symptomology have increased connectivity between the hippocampus and cingulate areas^99,100^, it is possible that our results provide a potential mechanistic window into how patients are able to overcome these effects during the prolonged prodromic phase of AD. It is possible that short spells of decreased oscillatory coherence are a neural adaptation that minimizes the cognitive impairments of increased connectivity. Perhaps, early in AD progression, patients, like hyperglycemic rats, can decrease oscillatory connectivity at times to perform well; however, over time, this ability fades as neurodegeneration progresses. This exciting possibility requires much more research; however, it does suggest that novel treatments which decrease connectivity might be effective. Overall, the counterintuitive coherence effects reported here in chronically hyperglycemic rats speaks to the serious impact that overly synchronized brain networks might have on cognition.

Understanding why network function was altered in STZ animals is a bit murkier, as there are multiple possible pathological aspects that may play a role, including tau hyperphosphorylation, altered blood glucose levels, and neuroinflammation. We have already discussed how increased tau levels may affect hippocampal and ACC activity, but we will also point out that it has recently been postulated that tauopathy impairs cognition when tau clusters at projection axons, thus impairing communication between areas^59^. It is also possible that the current results reflect the impact of altered insulin levels. Indeed, insulin levels in the brain are closely associated with object and spatial memory^101,102^, working memory performance^103^, and neurotransmitter signaling^104^. Impairments to insulin resistance can be induced by a high-fat diet leading to deficits in working memory in rodents^105^, and other forms of cognitive decline are found in prediabetic human patients exhibiting insulin resistance^106^. Concurrently, intracerebroventricular injections of STZ lead to phosphorylation of insulin receptors in the hippocampus and altered inflammatory signaling^9,107^, which impair insulin signaling in the brain. While previous findings have shown peripheral injections of STZ impair insulin-producing cells in the pancreas^108–110^, our results suggest that these effects impair neural circuitry and working memory. Future work is needed to disentangle how insulin levels affect network dysfunction and working memory impairments, where insulin levels are controlled after STZ treatment. Additionally, these manipulations will provide an intriguing model of DM patients who are in treatment.

The role of neuroinflammation is more poorly understood but ultimately might prove to be more relevant. Neuroinflammation is found in AD genetic models along with the tau or Aβ pathologies, but it is not known what effects are due to neuroinflammation as opposed to amyloidosis or tauopathy^111^. It has been shown that chronic neuroinflammation causes changes in hippocampal cells, such as altered ion channel dynamics^112^, excitability^113^, and synaptic integration^114^. Such cellular effects might potentially explain hippocampal network-level changes, but the mechanism is not clear. Furthermore, it is less clear how neuroinflammation might affect the ACC or interactions between the hippocampus and ACC, which are likely mediated by thalamic connections^55^. Intriguingly, abnormal delta activity from the thalamus into the hippocampus has been linked with impaired working memory retrieval^67^, perhaps hyperglycemia is affecting the thalamus leading to the increase in hippocampal delta power and altered hippocampal–ACC theta interactions. While more work is needed to better understand how neuroinflammation relates to network activity, there have been multiple reports revealing neuroinflammation-linked memory deficits in rodents^115,116^ and in humans^117^. A better understanding of how neuroinflammation, and the brain’s immune response in general, interacts with network activity is desperately needed and the current results document some network effects that need to be accounted for.

These experiments have revealed that STZ-induced hyperglycemia impairs hippocampal and ACC network activity. These experiments are a first step in understanding how hyperglycemia affects working memory network function and potentially explain why behavioral/cognitive deficits have been linked with DM^118,119^. The model and approach also provides an opportunity for investigations of the relationship of hyperglycemia as seen in DM and increased risk for AD. The changes in network function are similar to those found in AD patients and animal models, suggesting that there may be a more direct link between DM and AD than is currently known.

## Methods

### Subjects

Subjects were eight male Long-Evans rats (8–12 months) obtained from Charles River Laboratories, Inc. (Wilmington, MA), weighing between 400 and 550 g at the time of surgery and injections. Rats were on a restricted food intake of about 25 g per day while performing the behavioral task. The rats were singly housed and kept on a 12 h light:dark cycle. Training and recording sessions were carried out during the light cycle and were recorded roughly 3 days per week. All experimental procedures were approved by the University of Nevada Las Vegas Institutional Animal Care and Use Committee.

### Apparatus

We used a custom-built T-maze with three movable doors surrounding a delay area (see Fig. 1c). It was made using white, 14.5 cm wide textured plastic floors with ~28 cm high walls made from white corrugated plastic. The central stem of the T was 63.5 cm long connecting to the choice arms at the top of the stem totaling 162.5 cm. Each choice arm was roughly 74 cm in length with a small circular reward well near the end made of plastic piping 3 cm in diameter, 0.5 cm deep, and flush with the floor. A reservoir with a sugar-free chocolate beverage could be delivered to the reward wells through a small tube controlled by a photobeam tripped solenoid valve. The maze was elevated from the ground and surrounded by black curtains with distinctive visual cues attached. The maze and cues remained in the same location for the remainder of the experiment.

### Behavioral training

Prior to surgery and viral injections, rats were trained in multiple phases to perform a delayed spatial alternation task on a modified T-maze. Rats were first exposed to the maze to habituate to the new environment. Reward wells were baited with a sugary treat (Froot Loops; Kelloggs, Battlecreek, MI) to encourage exploration. Once acclimated, rats began the first phase of training that consisted of forced continuous alternating trials. Rats were placed in the sequester area of the maze with all doors closed. The center stem door opened, and rats would traverse to the end of the stem, where only one choice arm would be open on each trial. To encourage the return to the sequester area, the area was initially baited every trial and then periodically after returning became habitual. After animals were efficient at the forced alternation task (mean = 3 ± 2 sessions), trials were started with both choice arms open. In the second phase, the use of choice arm barriers was removed, allowing the rats to freely choose the correct arm. Animals were still rewarded with a sugary treat for correctly alternating between the two arms. In the third phase, rats were introduced to roughly 1 ml of a sugar-free chocolate beverage as a reward and required infrequent sequester baiting.

For all subsequent training and testing sessions, rats were placed in the enclosed sequester area with no choice arm barriers and allowed to run for 30–50 trials. The experimenter stayed outside the curtained enclosure for the duration of the session. Animal behavior was observed using a video monitor connected to a tracking system. On each trial with correct arm choice, a drop of a sugar-free chocolate beverage was delivered to the well in the arm following arm entry. Incorrect trials resulted in the end of a trial without a reward, and the rat was required to return to the sequester area on the correct return arm.

### Surgery and electrophysiology

Rats were anesthetized using isoflurane gas (1–3%) and surgically implanted with a 32-movable tetrode hyperdrive affixed to the animal’s skull. Tetrodes were made from a single 13 μm wire folded and wound to produce four independent channels. Of the 32 tetrodes, 16 were targeted bilaterally to the ACC (2.5 mm anterior; +0.5 lateral; 8 left and 8 right) and 16 bilaterally to dorsal CA1 (3.5 mm posterior to bregma; +2.5 lateral; 8 left and 8 right). Two screws were placed posterior, just above the cerebellum, used as grounding wires and soldered into the electrode interface board (EIB; Plexon Inc. Dallas, TX), typically done in rodent in vivo recordings^120^. Once tetrodes were positioned directly over the targeted brain areas, the implant was fixed in place using dental acrylic on top of the skull. After the dental acrylic hardened, tetrodes were lowered 400 mm into the cortex. Following a 7-day recovery period, tetrodes were then slowly lowered ventrally into the ACC (~2.5 mm, 10° angle) and the pyramidal cell layer of dorsal CA1 using known electrophysiological markers^120^. Each electrode was connected to a 128-channel EIB that could plug into four separate headstages (Intan Technologies, Los Angeles, CA). Electrophysiological signals were digitized and sent through two tethered cables into an RHD 2000 USB interface board (Intan Technologies, Los Angeles, CA), which is made visible through the OpenEphys (Cambridge, MA) interface. Data were sampled at a rate of 30 KHz. Continuous data were passed through a bandpass filter (0.1–6 KHz).

### Treatment groups

To induce a chronic hyperglycemic state, we used a modified STZ protocol, which consists of staggered and low doses of STZ injections^10^. Our goal was to achieve a chronic level of hyperglycemia with little changes in body weight. Due to the additional stress of neural implant surgery, we used a lower dose of STZ than Murtishaw et al.^10^. STZ (Sigma-Aldrich, MO, USA) was prepared fresh prior to administration by dissolving the drug in 0.1 M sodium citrate buffer (pH 4.5) for a final concentration of 20 mg/kg/ml. Following a 6-h fast, animals were injected with STZ via intraperitoneal injection. All injections were administered within 5 min of mixing, given that STZ is pharmacologically active for approximately 15 min^121^. Similarly, vehicle (citrate buffer) was administered to control groups by intraperitoneal injection (pH 4.5) at a final concentration of 20 mg/kg/ml. Our protocol to ensure that a sustained hyperglycemic state was based on achieving a group average fasting blood glucose level of ≥250 mg/dl consistent with DM levels. The modified protocol of staggered STZ injections on days 1, 2, 3, 14, and 15, followed by supplemental injections of STZ on days 35 and 36, achieved the criteria for sustained hyperglycemia consistent with Murtishaw et al.^10^.

### Tissue collection

Animals were euthanized via carbon dioxide asphyxiation. Animals dedicated for western blotting were trans-cardially perfused with 20 ml of sterile 1× phosphate-buffered saline (PBS). Hippocampal and cortical regions were quickly dissected and flash frozen with liquid nitrogen and stored at −80 °C until further tissue processing. Animals dedicated for histological analysis were initially trans-cardially perfused with 20 ml of sterile 1×PBS, followed by 20 ml of cold 4% sterile-filtered paraformaldehyde. Brains were removed and placed in 4% paraformaldehyde at 4 °C for 24 h. Afterwards, the brains were moved into a 10% sucrose solution for 24 h, 20% sucrose solution for 24 h, and stored in 30% sucrose solution + 0.05% sodium azide to prevent tissue contamination. Some tissue samples were unable to be included in this experiment due to problems with rodent implant detachment, thus, there was an *n* = 3 for both groups hippocampus and *n* = 3 for controls and *n* = 4 for STZ ACC for western blotting. All procedures and guidelines were followed in accordance to the Institutional Animal Care and Use Committee at the University of Nevada, Las Vegas.

### Protein extraction

Hippocampus and cortical regions were lysed for protein extraction by utilizing the BioPlex Cell Lysis Kit (BIO-RAD, CA, USA) with modifications. Briefly, 500 μl of complete cell lysis buffer was added to frozen tissue and mechanically homogenized (Kinematica Polytron 1300D, Luzern, CHE). The homogenates were then stored at −80 °C overnight. Frozen homogenates were then allowed to thaw on ice and immediately sonicated (Branson SFX 150, CT, USA), and centrifuged for 10 min at 10,000 × *g*, followed by supernatant collection. Protein concentrations were measured using the Pierce Bicinchoninic Acid Assay Kit (Thermo Fisher Scientific, MA, USA) with minor modifications. Protein lysates were diluted to 1000 ng/μl with a complete cell lysis buffer and stored at −80 °C until further processing.

### Western blot/sodium dodecyl sulfate–polyacrylamide gel electrophoresis (SDS-Page)

Proteins were loaded onto a 8% SDS-Page gel at a concentration of 10 μg and were separated by gel electrophoresis (Laemmli, 1970). Afterwards, the proteins were transferred onto a polyvinylidene difluoride transfer membrane (Cat. No. IPVH00010, Millipore-Sigma, MO, USA) followed by TBS-based blocking buffer (LI-COR, NE, USA) gently shaking overnight at 4 °C. On the following day, the membranes were incubated with primary antibody (rabbit anti-phospho-tau (pTau 396), 1:5000, Abcam ab109390; mouse anti-tau-5 (total tau), 1:5000, Millipore MAB361) gently shaking overnight in 4 °C. On the next day, the membranes were washed in 1×TBS + 0.1% Tween-20, followed by 1×TBS washes, and then incubated in IRDye near-infrared secondary antibodies (1:5000) for 1 h at room temperature. Lastly, the membranes were washed in 1×TBS + 0.1% Tween-20, followed by 1×TBS washes, and then imaged on ChemiDoc MP Imaging System (BIO-RAD, CA, USA). Target band intensity signals were quantified utilizing the Image Lab Software version 6.0 (BIO-RAD, CA, USA). Normalization of the target bands was completed using anti-total tau signals.

### Statistics and reproducibility

#### Behavioral analysis

To quantify behavioral changes that occurred during each session, we first examined running speed by linearizing the maze into 35 bins. We then calculated running speed by dividing total distance traveled between the two time points by the total time between the two same time points and then summed the total speed by each linearized bin. We then compared total distance traveled with one-way ANOVA between groups and speed over the maze with a two-factor ANOVA (Group × Maze Position). To test performance accuracy, trials were split into two roughly equally sized categories: short delays (<20 s) and long delays (>20 s). We then used a two-factor ANOVA (Group × Delay Length) to assess accuracy differences.

### Neural data analysis

#### LFP analysis

All analyses were performed in MATLAB (Mathworks, Natick, MA) using custom written scripts. LFP signals were analyzed from 4 leads in each area and each hemisphere (16 total). These leads were selected based on visual inspection in order to minimize noise. Only one wire from each tetrode could be selected. Signals were analyzed over the total of 35 sessions (19 STZ and 16 control). LFP signals were first notch filtered to remove 60 Hz noise. Spectral power was calculated with the *spectrogram* function in MATLAB. LFPs were sampled at 1 KHz and power spectral densities were computed over a window size of 1000 ms with 200 ms of overlap. Values from each area were then averaged, yielding one mean spectral power for the hippocampus and one for ACC for each session. Frequency band differences between groups were compared with ANOVAs.

#### SVM classification of area and group

For this analysis, first we calculated the spectral power over the entire session for each lead (16 per session). The power values (in proportion of total value) for the first 50 frequencies (1–50 Hz) were used for classification. We choose these frequencies to avoid introducing any bias that could be introduced by differing 60 Hz noise levels between sessions or animals. Next, we used the *fitceoc* function in MATLAB to construct SVMs to classify the location (ACC or hippocampus) and group (control or STZ) for each lead. Beforehand, we would randomly select a certain number of leads from each of the 4 classification groups (1—ACC control; 2—hippocampus control; 3—ACC STZ; 4—hippocampus STZ) to be the *training set* and the remaining leads were the *test set*. This process was repeated 1000 times for each analysis and the mean decoding accuracies are reported here. Our first analysis used iteratively larger numbers of leads in training sets starting with only 5 leads per classification group and moving by increments of 10 up to 85 lead training sets. We compared these values to 85 lead training sets with randomly assigned classification groups. This group/area shuffled comparison was also repeated 1000 times.

Next, we performed a leave one subject version of this analysis. This allowed us to see if the spectral power profiles were consistent across subjects. For this, we would randomly select 40 leads from each classification group from all the subjects except one to serve as the training set. We then used the remaining left out subject’s leads as the test set. This process was repeated 1000 times per subject and the mean decoding accuracies were reported.

Lastly, this same analysis was performed on individual sessions to ensure that the spectral profiles were consistent session to session. We randomly selected 40 leads from each classification group from all but one session and then used the remaining session as the test set. This process was repeated 1000 times for each session.

#### Cross-frequency coupling

PAC techniques were first described by Tort et al. For this analysis, we examined the modulation index (MI) or the rate at which the amplitude in certain higher frequency bands were influenced by the phase of lower frequency bands. All these analyses were performed on signals recorded from the same leads. MI values are between 0 and 1 and were generated using the freely available *Neurodynamics Lab* MATLAB toolbox function *ModIndex_v2*. MI values were calculated over the entire session. MI values were averaged for each area per session, yielding one ACC and one hippocampal MI value per session. Changes in MI between groups were examined using Kruskal–Wallis non-parametric ANOVAs.

#### ACC–hippocampus coherence

Coherence values were computed for each possible pair of ipsilateral ACC and hippocampus leads using the *mscohere* function in MATLAB. The coherence value is computed as:

$${C}_{xy}\left(f\right)=\frac{{{{\rm{|}}}}{{P}_{xy}\left(f\right){{{\rm{|}}}}}^{2}}{{P}_{xx}\left(f\right){P}_{yy}\left(f\right)}$$

For the analysis in Fig. 5, coherence values were calculated separately for each trial. These values were then averaged over the last 10 s of the delay period and the first 2 s of the trial run for the comparisons shown in Fig. 5. Then the mean values from all ipsilateral recording lead combinations were computed per session or trial. Coherence values between correct and error trials were compared with non-parametric Kruskal–Wallis ANOVAs.

### Reporting summary

Further information on research design is available in the Nature Research Reporting Summary linked to this article.

## Supplementary information

Supplementary Information

Description of Additional Supplementary Files

Supplementary Data 1

Reporting Summary

## Data Availability

The electrophysiological and behavioral data that support the findings of this study are available by request from the corresponding author. The source data for the protein assays are shown in Supplementary Data 1.

## Code availability

All code are available by request from the corresponding author.
