# Supplementary material for: Altered theta rhythm and hippocampal-cortical interactions underlie working memory deficits in a hyperglycemia risk factor model of Alzheimer’s disease
Source: Commun Biol. 2021 Sep 3;4:1036. doi: 10.1038/s42003-021-02558-4 (PMC8417282; doi:10.1038/s42003-021-02558-4)

Supplemental Information

Supplemental Figure 1: Merged western blot image of pTau396/Tau. Black rectangular outline on membrane indicates protein kDa used for analysis based on the manufactures predicted band (i.e., pTau396 = 50-70kDa; Tau = 45-68kDa). BIO-RAD Plus Protein All Blue Standards seen in channel IRDye 680RD was utilized as a ladder to determine approximate band size. BIO-RAD ChemiDoc MP Imaging System and Image Lab Software were used to image and analyze band intensities, respectively.

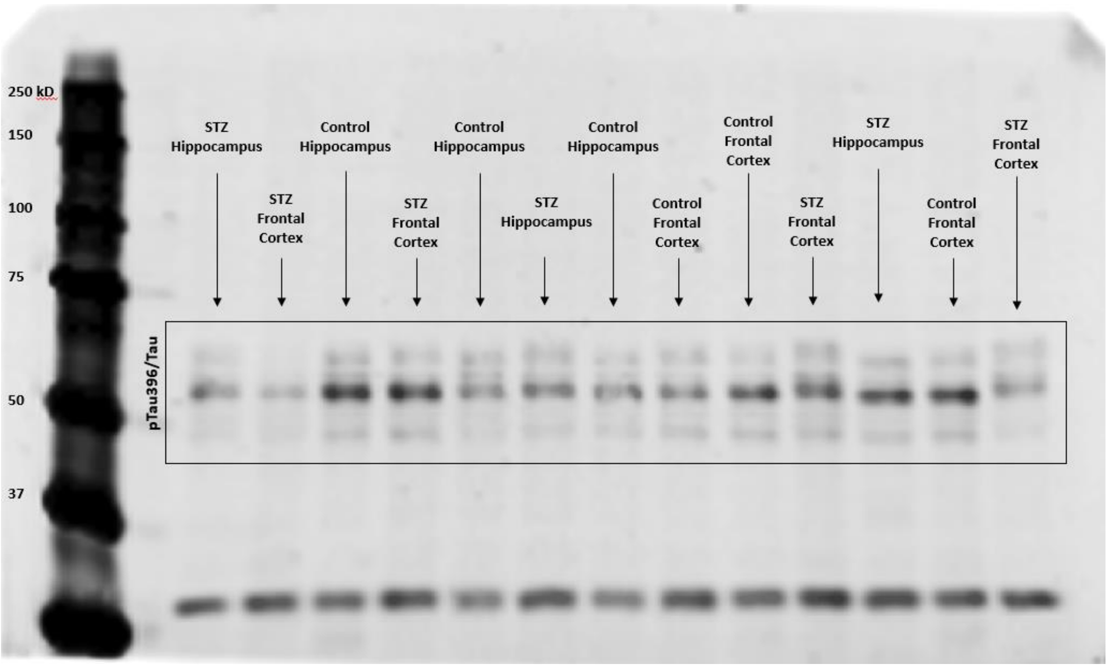

Supplemental Figure 1a: Western blot image of channel IRDye 800CW (pTau396). Black rectangular outline on membrane indicates protein kDa used for analysis based on the manufactures predicted band (i.e., pTau396 = 50-70kDa). No ladder was observed in channel IRDye 800CW. Instead, ladder from channel IRDye 680RD seen in supplemental figure 1 (i.e., merged image of pTau396/Tau) was utilized to determine approximate band size. BIO-RAD ChemiDoc MP Imaging System and Image Lab Software were used to image and analyze band intensities, respectively.

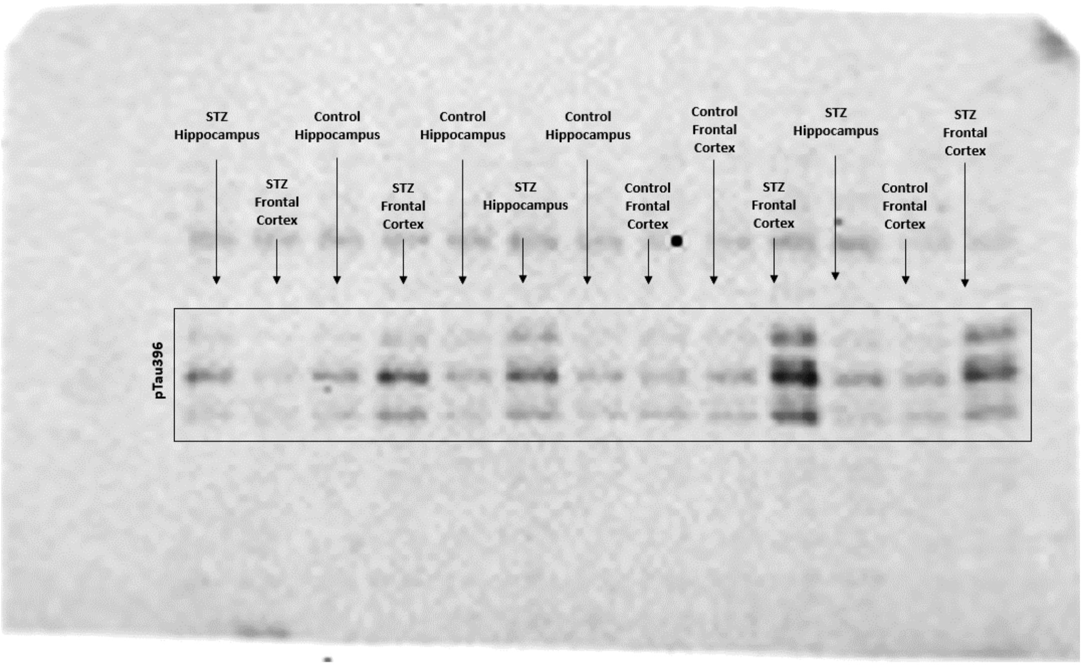

Supplemental Figure 1b: Western blot image of channel IRDye 680RD (Tau). Black rectangular outline on membrane indicates protein kDa used for analysis based on the manufactures predicted band (i.e., Tau = 45-68kDa). BIO-RAD Plus Protein All Blue Standards was utilized as a ladder to determine approximate band size. BIO-RAD ChemiDoc MP Imaging System and Image Lab Software were used to image and analyze band intensities, respectively.

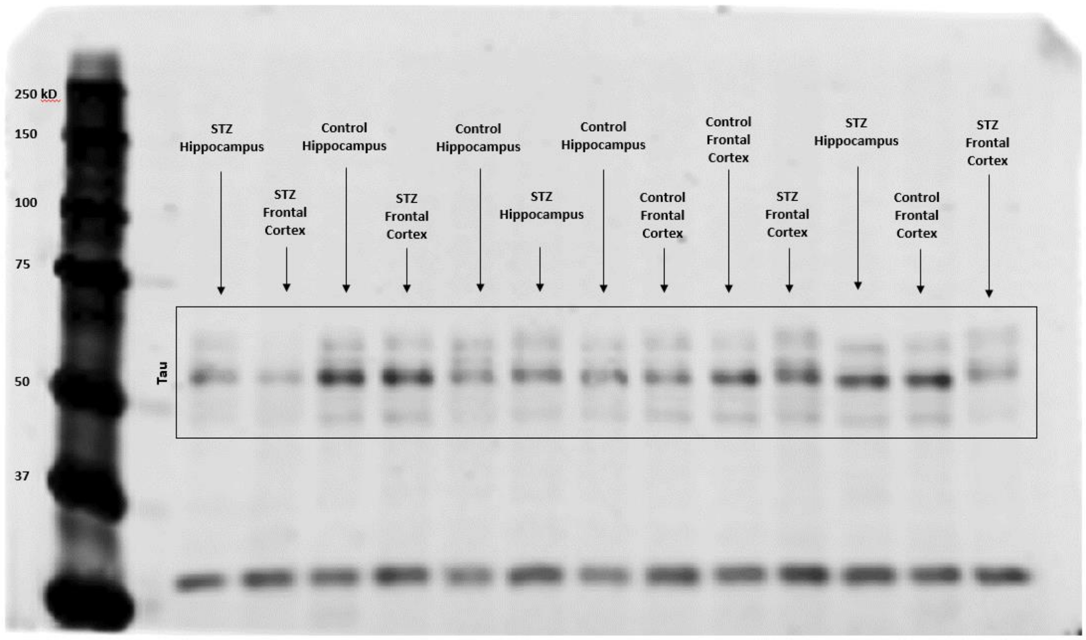

Supplemental Figure 2: Merged western blot image of pTau396/Tau for sample replicate two. Black rectangular outline on membrane indicates protein kDa used for analysis based on the manufactures predicted band (i.e., pTau396 = 50-70kDa; Tau = 45-68kDa). BIO-RAD Plus Protein All Blue Standards seen in channel IRDye 680RD was utilized as a ladder to determine approximate band size. BIO-RAD ChemiDoc MP Imaging System and Image Lab Software were used to image and analyze band intensities, respectively.

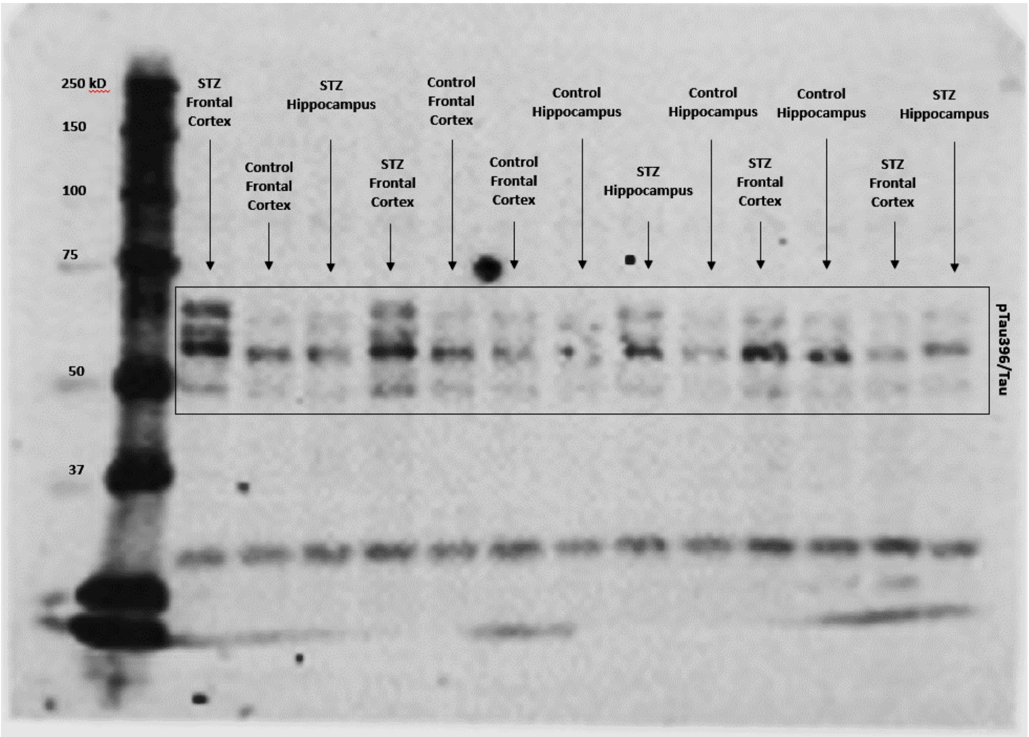

Supplemental Figure 2a: Western blot image of channel IRDye 800CW (pTau396) for sample replicate two. Black rectangular outline on membrane indicates protein kDa used for analysis based on the manufactures predicted band (i.e., pTau396 = 50-70kDa). No ladder was observed in channel IRDye 800CW. Instead, ladder from channel IRDye 680RD seen in supplemental figure 2 (i.e., merged image of pTau396/Tau) was utilized to determine approximate band size. BIO-RAD ChemiDoc MP Imaging System and Image Lab Software were used to image and analyze band intensities, respectively.

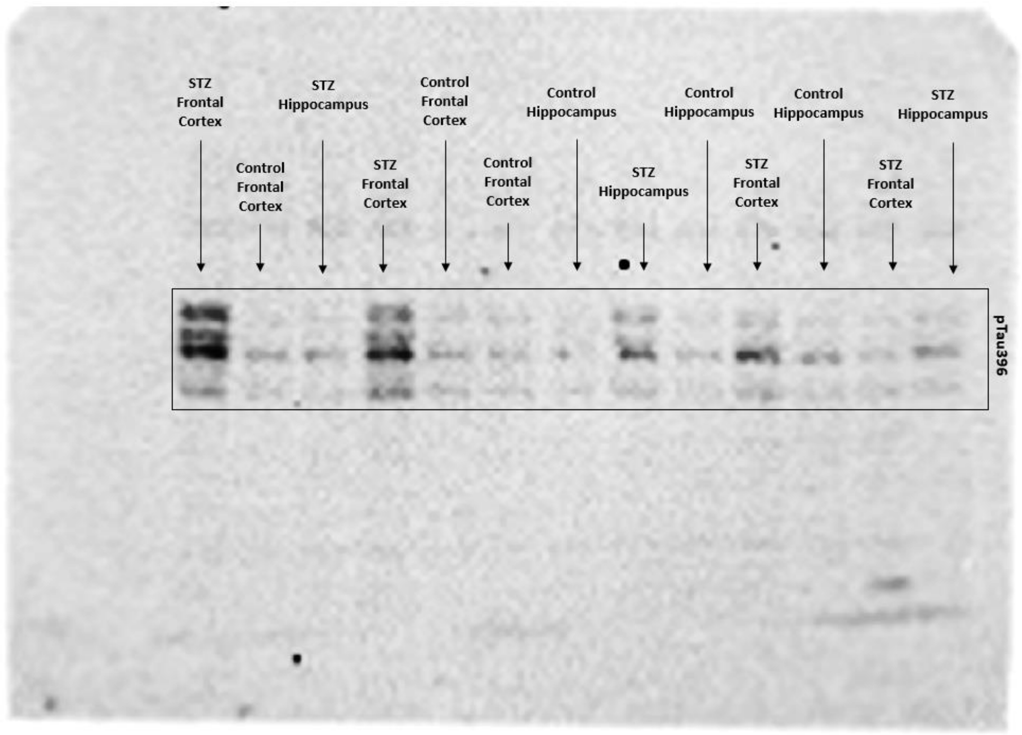

Supplemental Figure 2b: Western blot image of channel IRDye 680RD (Tau) for sample replicate two. Black rectangular outline on membrane indicates protein kDa used for analysis based on the manufactures predicted band (i.e., Tau = 45-68kDa). BIO-RAD Plus Protein All Blue Standards was utilized as a ladder to determine approximate band size. BIO-RAD ChemiDoc MP Imaging System and Image Lab Software were used to image and analyze band intensities, respectively.

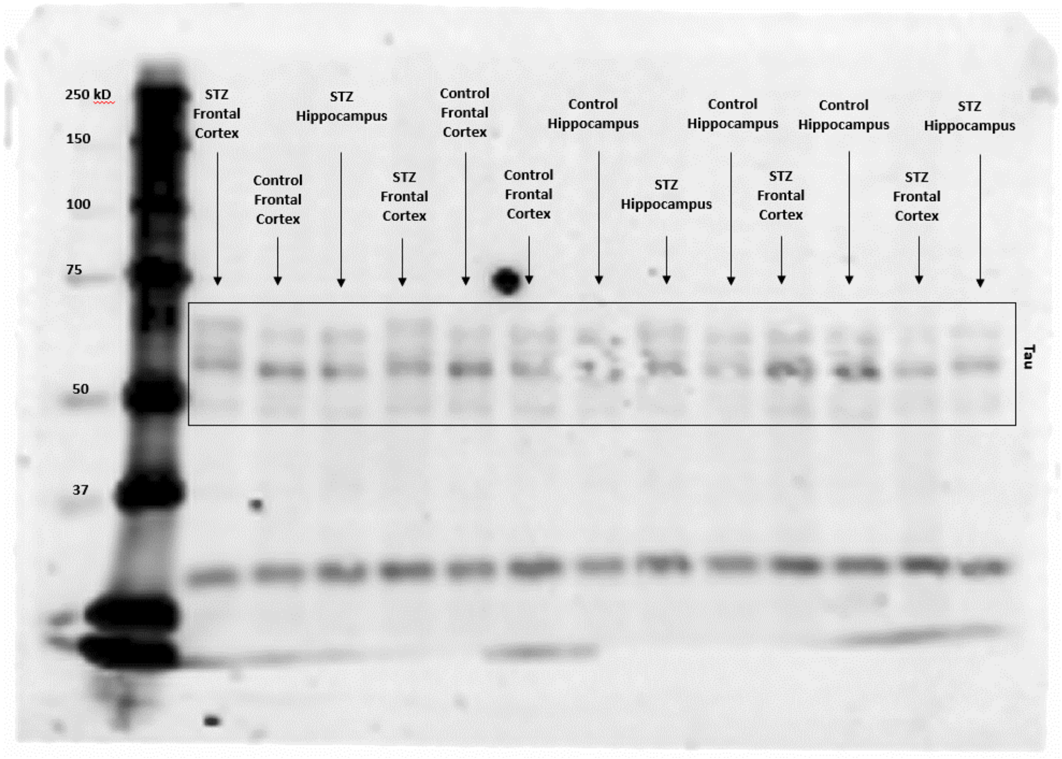

Supplemental Figure 3: Merged western blot image of pTau396/Tau for sample replicate three. Black rectangular outline on membrane indicates protein kDa used for analysis based on the manufactures predicted band (i.e., pTau396 = 50-70kDa; Tau = 45-68kDa). BIO-RAD Plus Protein All Blue Standards seen in channel IRDye 680RD was utilized as a ladder to determine approximate band size. BIO-RAD ChemiDoc MP Imaging System and Image Lab Software were used to image and analyze band intensities, respectively.

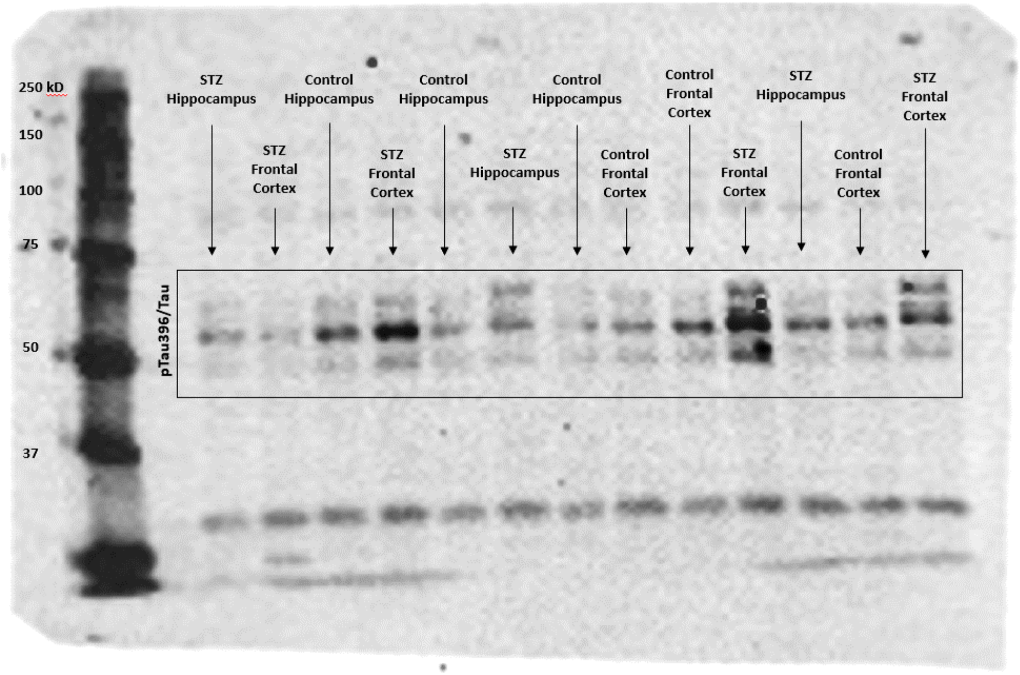

Supplemental Figure 3a: Western blot image of channel IRDye 800CW (pTau396) for sample replicate three. Black rectangular outline on membrane indicates protein kDa used for analysis based on the manufactures predicted band (i.e., pTau396 = 50-70kDa). No ladder was observed in channel IRDye 800CW. Instead, ladder from channel IRDye 680RD seen in supplemental figure 3 (i.e., merged image of pTau396/Tau) was utilized to determine approximate band size. BIO-RAD ChemiDoc MP Imaging System and Image Lab Software were used to image and analyze band intensities, respectively.

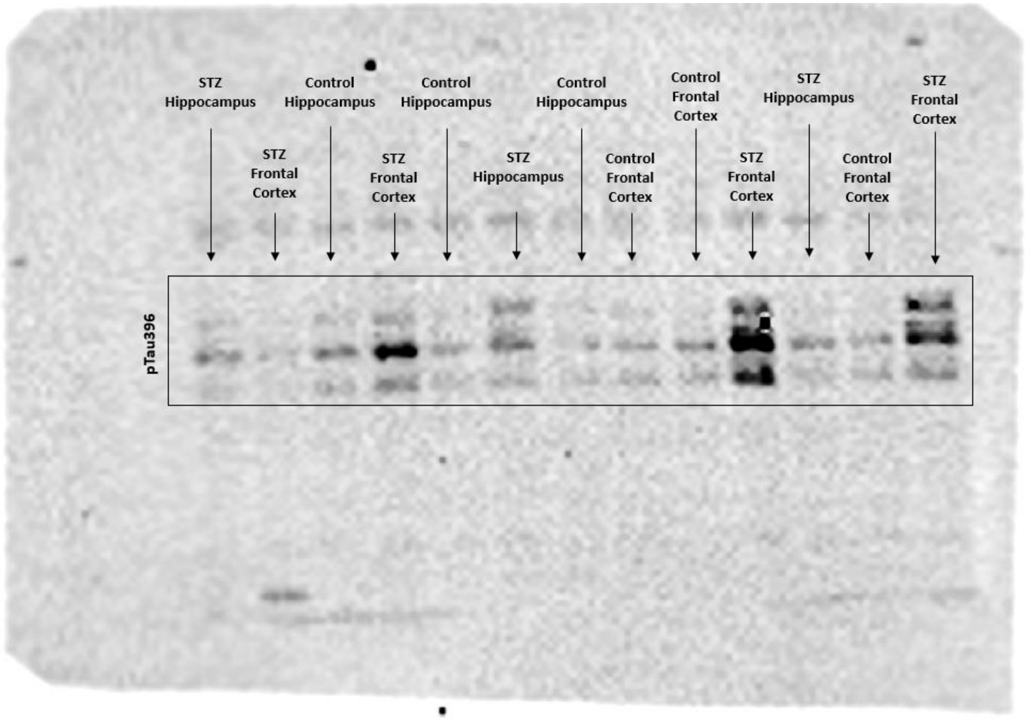

Supplemental Figure 3b: Western blot image of channel IRDye 680RD (Tau) for sample replicate three. Black rectangular outline on membrane indicates protein kDa used for analysis based on the manufactures predicted band (i.e., Tau = 45-68kDa). BIO-RAD Plus Protein All Blue Standards was utilized as a ladder to determine approximate band size. BIO-RAD ChemiDoc MP Imaging System and Image Lab Software were used to image and analyze band intensities, respectively.

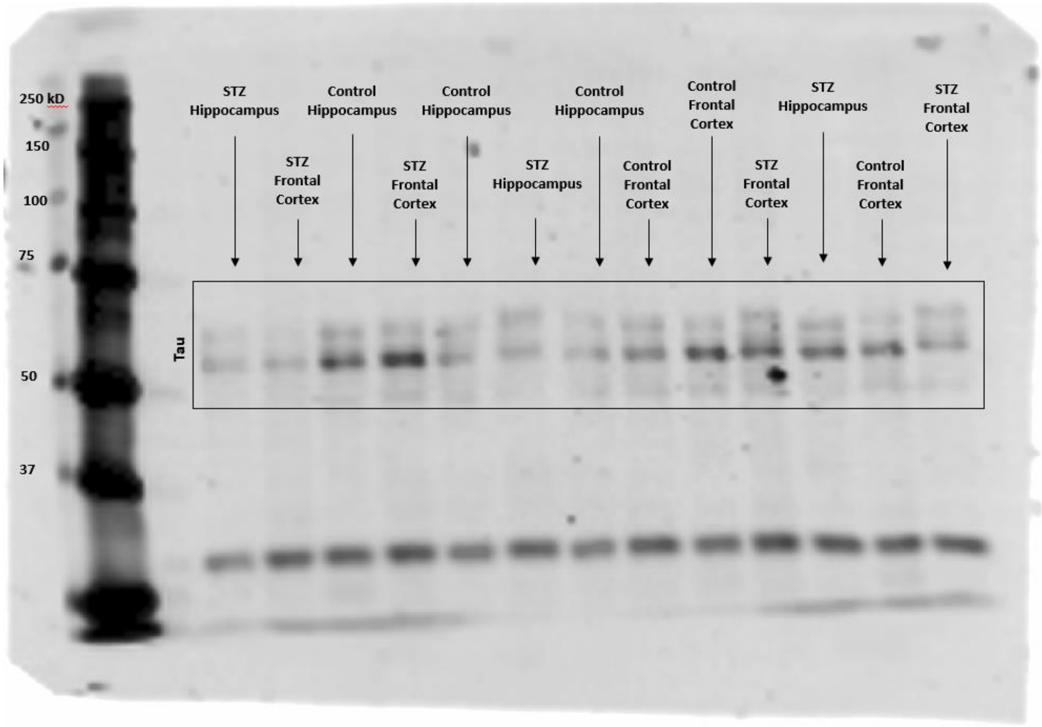

Supplemental Figure 4: Merged western blot image of pTau396/Tau for sample replicate four. Black rectangular outline on membrane indicates protein kDa used for analysis based on the manufactures predicted band (i.e., pTau396 = 50-70kDa; Tau = 45-68kDa). BIO-RAD Plus Protein All Blue Standards seen in channel IRDye 680RD was utilized as a ladder to determine approximate band size. BIO-RAD ChemiDoc MP Imaging System and Image Lab Software were used to image and analyze band intensities, respectively. Note: wells 8 and 9 labeled "removed from analysis" were removed from analysis due to high intensity particles on target band for both channels (i.e., IRDye 800CW and IRDye 680RD).

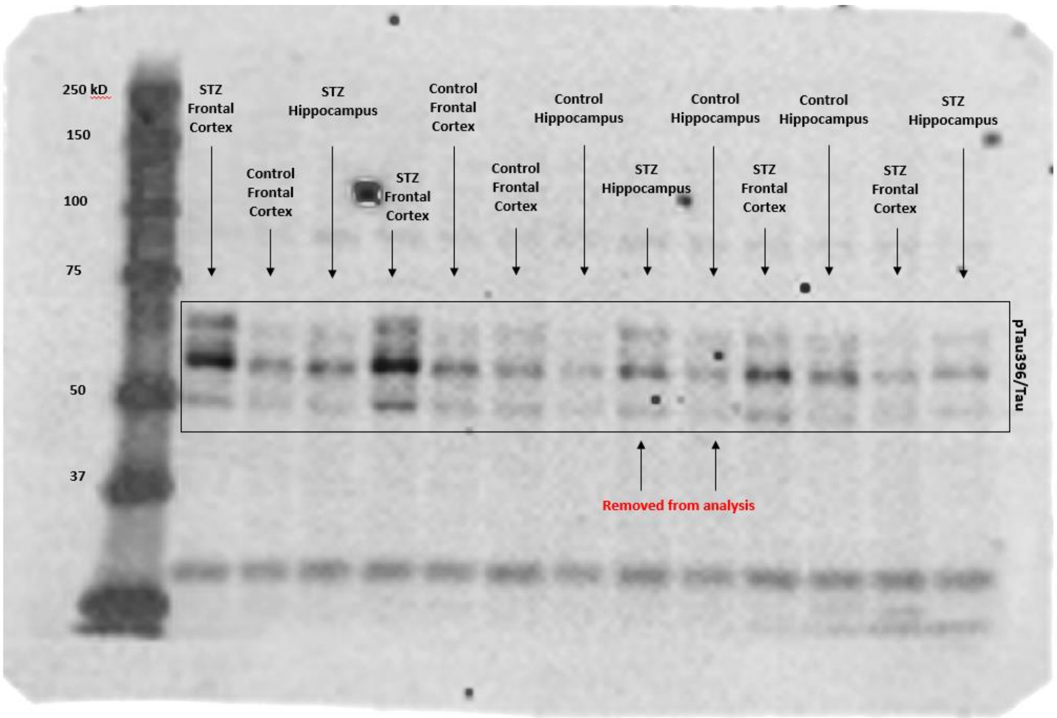

Supplemental Figure 4a: Western blot image of channel IRDye 800CW (pTau396) for sample replicate four. Black rectangular outline on membrane indicates protein kDa used for analysis based on the manufactures predicted band (i.e., pTau396 = 50-70kDa). No ladder was observed in channel IRDye 800CW. Instead, ladder from channel IRDye 680RD seen in supplemental figure 4 (i.e., merged image of pTau396/Tau) was utilized to determine approximate band size. BIO-RAD ChemiDoc MP Imaging System and Image Lab Software were used to image and analyze band intensities, respectively. Note: wells 8 and 9 were removed from analysis due to high intensity particles on target band.

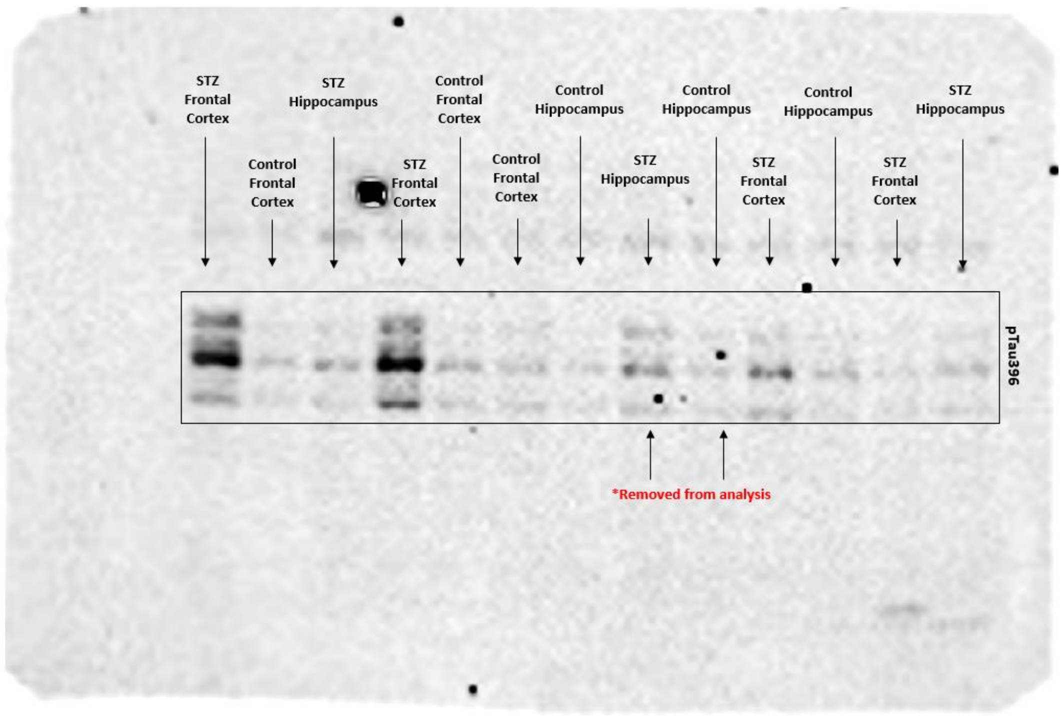

Supplemental Figure 4b: Western blot image of channel IRDye 680RD (Tau) for sample replicate four. Black rectangular outline on membrane indicates protein kDa used for analysis based on the manufactures predicted band (i.e., Tau = 45-68kDa). BIO-RAD Plus Protein All Blue Standards was utilized as a ladder to determine approximate band size. BIO-RAD ChemiDoc MP Imaging System and Image Lab Software were used to image and analyze band intensities, respectively. Note: wells 8 and 9 were removed from analysis due to high intensity particles on target band.

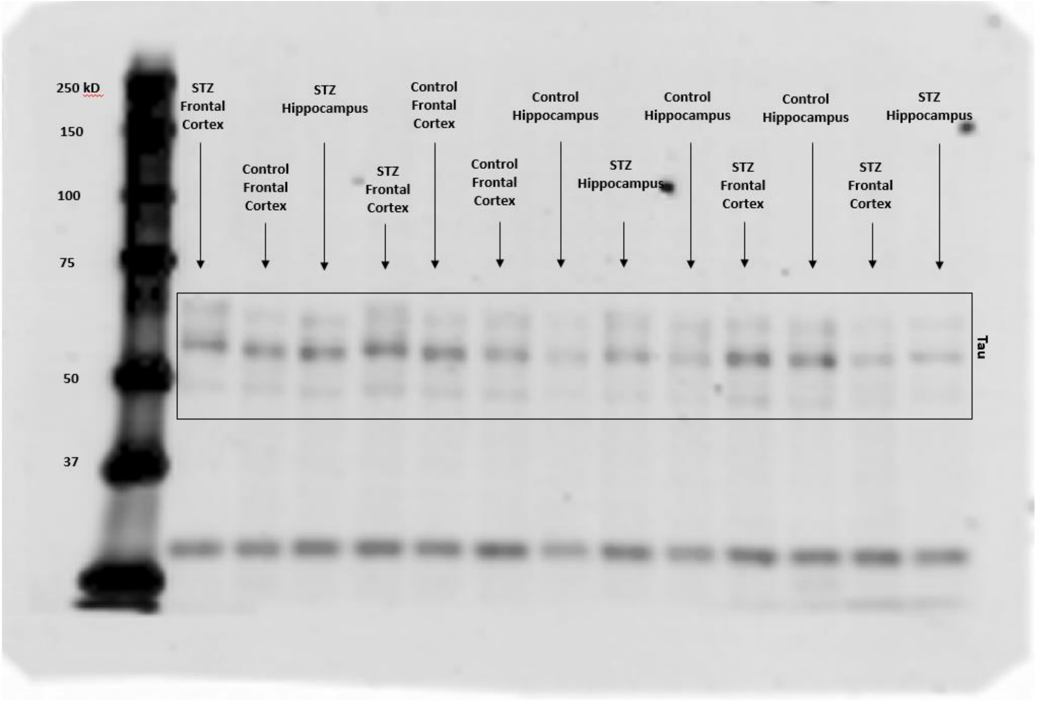

Supplement: Supplementary file 1 — Supplementary Information [file 42003_2021_2558_MOESM1_ESM.pdf]
