# Supplementary material for: Altered theta rhythm and hippocampal-cortical interactions underlie working memory deficits in a hyperglycemia risk factor model of Alzheimer’s disease
Source: Commun Biol. 2021 Sep 3;4:1036. doi: 10.1038/s42003-021-02558-4 (PMC8417282; doi:10.1038/s42003-021-02558-4)
Supplement: Supplementary file 2 — Description of Additional Supplementary Files [file 42003_2021_2558_MOESM2_ESM.pdf]

Description of Additional Supplementary Files

File name: Supplementary Data 1

Description: Western blot images of pTau396/Tau. Each sample had four replicates on four independent blots (i.e., membranes 1, 2, 3, and 4). Each membrane contains a merged image of pTau396/Tau, as well as each separate channel (i.e., channel 800 = pTau396; channel 680 = Tau). Black rectangular outline on each membrane indicates protein kDa used for analysis based on the manufactures predicted band (i.e., pTau396 = 50-70kDa; Tau = 45-68kDa). Two samples on membrane 4 were excluded from analysis due to high intensity particles. BIO-RAD ChemiDoc MP Imaging System and Image Lab Software were used to image and analyze intensities, respectively.
